# Liver-Metastasis-Related Genes are Potential Biomarkers for Predicting the Clinical Outcomes of Patients with Pancreatic Adenocarcinoma

**DOI:** 10.3389/pore.2021.1609822

**Published:** 2021-07-05

**Authors:** Yinlei Dong, Junjie Tian, Bingqian Yan, Kun Lv, Ji Li, Deliang Fu

**Affiliations:** ^1^ Department of Pancreatic Surgery, Pancreatic Disease Institute, Huashan Hospital, Fudan University, Shanghai, China; ^2^ Department of Urology, The First Affiliated Hospital, School of Medicine, Zhejiang University, Hangzhou, China; ^3^ Children’s Hospital of Fudan University, National Children’s Medical Center, Shanghai, China; ^4^ Department of Radiology, Huashan Hospital, Fudan University, Shanghai, China

**Keywords:** overall survival, pancreatic cancer, nomogram, liver metastasis, prognostic signature, R0 resection

## Abstract

It is widely acknowledged that metastasis determines the prognosis of pancreatic adenocarcinoma (PAAD), and the liver is the most primary distant metastatic location of PAAD. It is worth exploring the value of liver-metastasis-related genetic prognostic signature (LM-PS) in predicting the clinical outcomes of PAAD patients post R0 resection. We collected 65 tumors and 165 normal pancreatic data from The Cancer Genome Atlas (TCGA) and the Genotype-Tissue Expression project (GTEx), respectively. Differentially expressed genes (DEGs) between primary tumor and normal pancreatic samples were intersected with DEGs between primary tumor samples with liver metastasis and those without new tumor events. The intersected 45 genes were input into univariate Cox regression analysis to identify the prognostic genes. Thirty-three prognostic liver-metastasis-related genes were identified and included in least absolute shrinkage and selection operator (LASSO) analysis to develop a seven-gene LM-PS, which included six risk genes (ANO1, FAM83A, GPR87, ITGB6, KLK10, and SERPINE1) and one protective gene (SMIM32). The PAAD patients were grouped into low- and high-risk groups based on the median value of risk scores. The LM-PS harbored an independent predictive ability to distinguish patients with a high-risk of death and liver metastasis after R0 resection. Moreover, a robust prognostic nomogram based on LM-PS, the number of positive lymph nodes, and histologic grade were established to predict the overall survival of PAAD patients. Besides, a transcription factor‐microRNA coregulatory network was constructed for the seven LM-PS genes, and the immune infiltration and genomic alterations were systematically explored in the TGCA-PAAD cohort.

## Introduction

Pancreatic adenocarcinoma (PAAD) is one of the most lethal malignancies with strong metastatic ability. It is predicted to become the second most common cause of cancer-related death within the next decade [1,2]. Despite advances in diagnostic techniques, radiotherapies and systemic therapies for PAAD, the five-years overall survival (OS) remains 10%, because 80–85% of the patients are initially diagnosed with either unresectable or metastatic tumors [3,4]. For the small fraction of the patients with a resectable and localized tumor, the five-years survival rate post-operation was 20% [3]. It is widely accepted that metastasis determines the prognosis of patients with pancreatic cancer [5]. Compared with patients with localized tumors, the survival of patients with metastasis is only 6–8 months [6]. The most primary distant metastatic location of PAAD is the liver [7]. Therefore, it is meaningful to explore the potential biomarkers with the ability to distinguish patients with unfavorable prognosis and high-risk of liver metastasis.

The hepatic metastasis process of PAAD involves complicated steps such as the adhesion of dissociated pancreatic cancer cells toward the liver, the formation of the remodeled extra-cellular matrix (ECM), the angiogenesis for micro-metastasis, and the construction of immune escape [7]. Previous studies have revealed that pancreatic tumors are highly heterogeneous at both cellular and molecular levels [8,9]. Molecular biomarkers play increasingly important roles in predicting the prognosis of patients with PAAD [10]. Multiple prognostic models with reliably predictive value have been established based on the mining of public databases such as The Cancer Genome Atlas (TCGA), International Cancer Genome Consortium (ICGC), and Gene Expression Omnibus (GEO) [11–13]. Recently, Venkat et al. reported that the alternative polyadenylation promoted the expression of the protumorigenic gene in pancreatic ductal adenocarcinoma (PDAC) by mining the integrated data of the Genotype-Tissue Expression (GTEx) project and TCGA [14]. There are few studies on mRNA combination biomarkers for the liver metastasis of PAAD. In the current study, we hypothesized that the differentially expressed genes associated with liver metastasis might harbor the potential for predicting the prognosis of patients with PAAD.

In this study, we integrated the mRNA expression data of normal pancreas and PAAD tissues from the GTEx and the TCGA datasets, respectively. A seven-gene prognostic signature was constructed with the ability to predict both OS and liver metastasis for PAAD patients following R0 resection.

## Materials and Methods

### Patient Selection and Data Retrieval

Patient inclusion criteria in the TCGA dataset were [1] PAAD patients with a surgical operation of R0 resection [2], patients with no new tumor event or with liver metastasis during follow-up. The exclusion criteria were [1] patients with survival period <30 days or with incomplete survival data [2], patients with missing data of gene expression. Sixty-five patients were included in this study. During the follow-up period, the numbers of patients with liver metastasis and with no new tumor event were 15 and 50, respectively. The corresponding clinicopathological information of the PAAD patients was obtained *via* Genomic Data Commons Data Portal (https://portal.gdc.cancer.gov/). The mRNA expression data [Fragments Per Kilobase of exon model per Million mapped fragments (FPKM)] of the PAAD samples in the TCGA dataset and the normal pancreas samples in the GTEx dataset were reprocessed using UCSC xena (https://xenabrowser.net/) to avoid data imbalance and transformed into log_2_(FPKM+1). The batch effects between the TCGA and the GTEx cohorts were minimized by “limma” R package. To assess the batch effects, the variation of housekeeping gene expression between the TCGA and the GTEx datasets was evaluated as Eisenberg et al. reported [15]. The mRNA expression profiles of the 65 PAAD and 165 normal pancreas samples were obtained for downstream analysis. The GRCh38 file was downloaded from the Ensembl website (https://asia.ensembl.org/index.html) for annotation of the mRNAs.

The 65 PAAD patients in the TCGA cohort were training dataset. The whole TCGA-PAAD dataset with 147 patients (cases with a survival period ≥30 days and complete gene expression data), GEO (https://www.ncbi.nlm.nih.gov/geo/) and ICGC (obtained from SurvExpress website, http://bioinformatica.mty.itesm.mx:8080/Biomatec/SurvivaX.jsp) were used as validation datasets.

### Identification of Differentially Expressed Genes Associated with Liver Metastasis

Based on the threshold of |logFC| > 1 and *p* value <0.05, the differentially expressed genes (DEGs) of normal pancreas samples vs. primary tumor samples, and DEGs of primary tumor samples without new tumor event vs. primary tumor samples with liver metastasis during follow-up were screened by Wilcoxon test. Venn diagram tool (http://bioinformatics.psb.ugent.be/webtools/Venn/) was used to identify the intersection of DEGs in PAAD tissues and PAAD tissues with liver metastasis.

Prognostic Gene Signature Construction Based on Liver-metastasis-related mRNAs.

Univariate regression Cox analysis was performed to filter mRNAs associated with OS. Then the identified mRNAs were included in the least absolute shrinkage and selection operator (LASSO) Cox regression model to develop a liver-metastasis-related prognostic signature (LM-PS) for the PAAD patients involving seven liver-metastasis-related genes and derive the regression coefficient of each gene by “glmnet” and “survival” R packages. Thereafter, risk scores for patients were calculated according to the following formula:

$$\begin{array}{l} Risk\;score=\overset{n}{\underset{i=1}{\sum }}\left(Coef_{i}*Exp_{i}\right) \end{array}$$

where $Exp_{i}$ represents the expression of each prognostic mRNA in the LM-PS, and $Coef_{i}$ represents the coefficient of the corresponding mRNA. PAAD patients were grouped into low- and high-risk groups based on the median value of the risk scores. Principal component analysis (PCA) was performed for the two groups using the R package “factoextra”.

### Independence of the Liver-Metastasis-Related Prognostic Signature for Predicting Overall Survival and Liver Metastasis

In the TCGA dataset, the liver-metastasis-related prognostic signature and corresponding clinicopathological factors of the PAAD patients were included in univariate/multivariate Cox proportional hazards analysis and logistic regression model analysis to identify the independent predictors of OS and liver metastasis, respectively. A nomogram was established based on the independent predictors of OS with the “rms” R package. The receiver operating characteristic (ROC) curves and the area under the curve (AUC) values were used to evaluate the predictive ability of the established models in this study.

### Functional Enrichment Analysis

Based on the threshold of |logFC| > 1 and *p* value <0.05, 408 DEGs between high- and low-risk groups were identified and uploaded to the “Metascape” website (https://metascape.org/) for enrichment analysis involving Gene Ontology (GO) and the Kyoto Encyclopedia of Genes and Genomes Pathway (KEGG).

### The Immune Infiltration Analysis of PAAD

The infiltration levels of the 22 immune cells were determined to assess the tumor microenvironment between high- and low-risk groups and between liver-metastasis and non-new-tumor groups in the TGCA dataset by the CIBERSORT R script v1.03. A *p* value <0.05 was set as the significance threshold.

### Gene Mutation Profiles of the PAAD Patients in the TCGA Dataset

The cBioPortal website (http://www.cbioportal.org/) is a large depository that provides access to cancer genomics data carried out by many institutions including the TCGA database [16]. In this study, the cBioPortal was used to explore the connection between the gene mutation and the prognosis of the 65 PAAD patients in the TCGA dataset.

### Transcription Factor‐microRNA Coregulatory Network Construction

The seven liver-metastasis-related genes identified by the Lasso Cox regression model were uploaded to NetworkAnalyst (https://www.networkanalyst.ca/faces/home.xhtml) to construct a transcription factor‐microRNA coregulated network. The transcription factor‐microRNA coregulatory data were predicted from the Regulatory Network Repository. The transcription factor‐microRNA coregulatory network was visualized by Cytoscape 3.8.1.

### Statistical Analysis

PAAD patients in the TCGA cohort were divided into high- and low-risk groups based on the median value of the risk scores. Mean ± standard deviation or medians (with interquartile range), and counts with percentages were used to present continuous variables and categorical variables, respectively. Continuous variables were compared by Student’s *t*-test or Mann-Whitney *U*-test, and categorical variables by Chi-square test. Kaplan-Meier curves were plotted to analyze patient survival. The log-rank tests were performed to analyze the differences in patient survival. The statistical analysis was carried out with the R software 4.0.2, SPSS 22.0, and GraphPad Prism 8.0.1. *p* values <0.05 were considered to indicate statistical significance (two-sided).

## Results

### Identification of Liver-Metastasis-Related Genes in PAAD Patients

**FIGURE 1:**
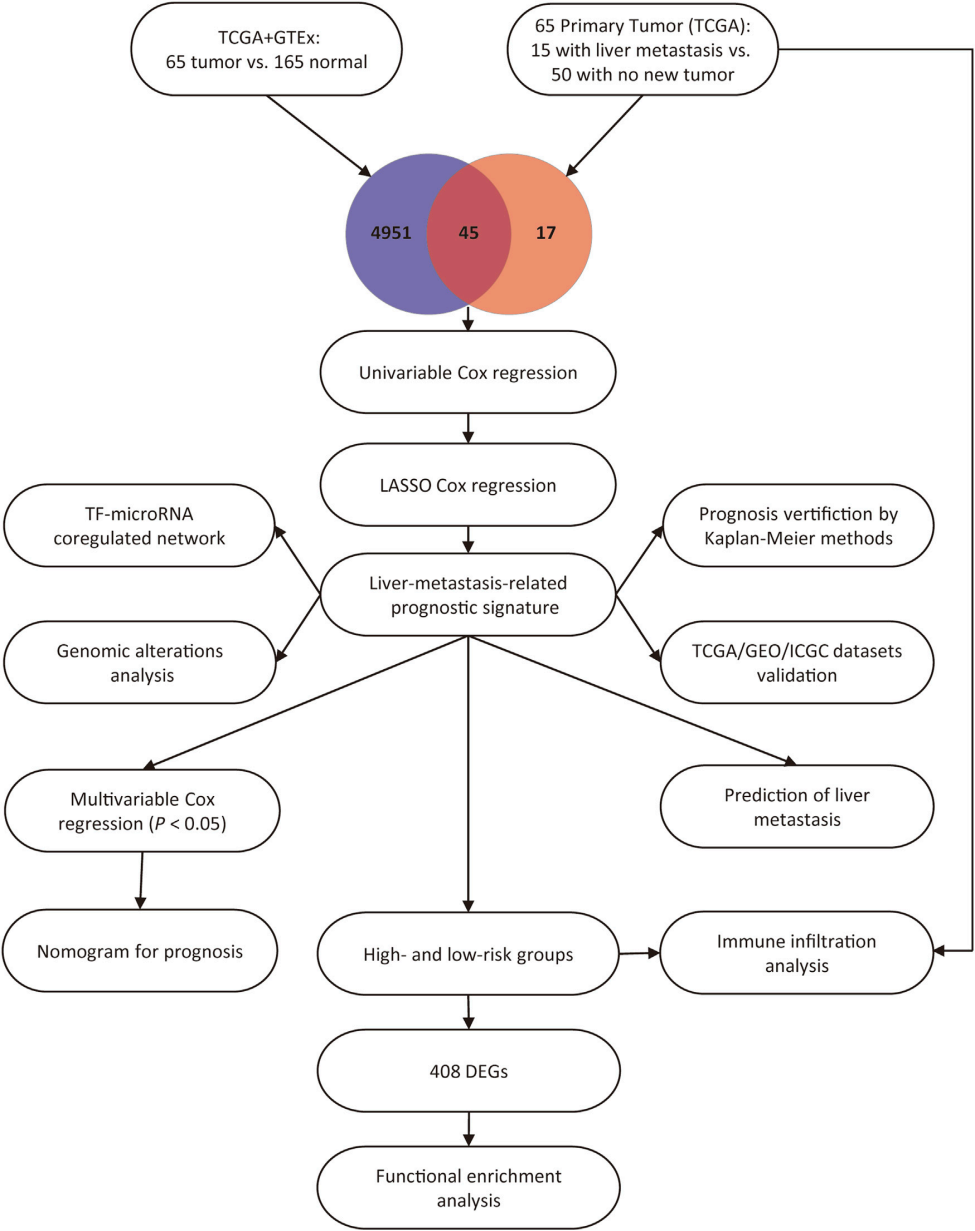
Study flow chart.

This study included 65 PAAD patients with R0 resection from the TCGA dataset, and the corresponding clinicopathological factors of the patients were presented in Table 1. As shown in the table, with the exception of survival status and pathologic M stage, there was no significant difference in terms of all the other clinicopathological variables that were analyzed. Previous studies successfully compared the gene expression between the GTEx and TCGA datasets, and minimized the batch effects in data processing [14,17]. To assess the batch effects in this study, the variation of housekeeping genes expression between the TCGA and GTEx datasets was compared, and a high correlation between the PAAD and normal pancreas tissues was observed (Pearson R = 0.89, *p* < 0.0001, Supplementary Figure S1), indicating minimal batch effects between the two datasets. Subsequently, we screened DEGs based on the standards of |logFC| > 1 and *p* < 0.05 between the 65 PAAD tissues vs. 165 normal pancreas tissues and between the 15 primary PAAD tissues with liver metastasis vs. 50 primary PAAD tissues without new tumor event. The results showed that 4,996 and 62 genes were differently expressed in PAAD tissues and PAAD tissues with liver metastasis, respectively. Thereafter, the Venn diagram web tool was used to get the intersection of the two groups of DEGs, and 45 liver-metastasis-related genes were identified. The study flow chart is shown in Figure 1.

**TABLE 1:** Clinicopathological factors of the PAAD patients in TCGA dataset.

Clinical features	Patients with no new tumor (n = 50)	Patients with liver metastasis (n = 15)	*p*
Age at diagnosis (years), mean ± SD	61.16 ± 11.1	62.9 ± 9.3	0.577
Gender, n (%)			0.548
Female	19 (38)	7 (46.7)	
Male	31 (62)	8 (53.3)	
Number of positive lymph nodes, median (interquartile range)	1 (0–3)	2 (0-5)	0.357
Survival status, n (%)			<0.001
Alive	40 (80.0)	3 (20.0)	
Dead	10 (20.0)	12 (80.0)	
Histologic grade, n (%)			0.473
G1	11 (22.0)	2 (13.3)	
G2	23 (46.0)	5 (33.3)	
G3	14 (28.0)	7 (46.7)	
G4	1 (2.0)	0	
GX	1 (2.0)	1 (6.7)	
Pathologic stage, n (%)			0.746
Stage I	8 (16.0)	1 (6.7)	
Stage II	39 (78.0)	14 (93.3)	
Stage III	0	0	
Stage IV	1 (2.0)	0	
Not available	2 (4.0)	0	
Pathologic T stage, n (%)			0.441
T1	5 (10.0)	0	
T2	8 (16.0)	1 (6.7)	
T3	35 (70.0)	14 (93.3)	
TX	2 (4.0)	0	
Pathologic N stage, n (%)			1.000
N0	16 (32.0)	5 (33.3)	
N1	31 (62.0)	10 (66.7)	
NX	3 (6.0)	0	
Pathologic M stage, n (%)			0.019
M0	17 (34.0)	11 (73.3)	
M1	1 (2.0)	0	
MX	32 (64.0)	4 (26.7)	

PAAD, pancreatic adenocarcinoma; TCGA, The Cancer Genome Atlas; SD, standard deviation.

### Construction of the LM-PS in the TCGA Cohort

**FIGURE 2:**
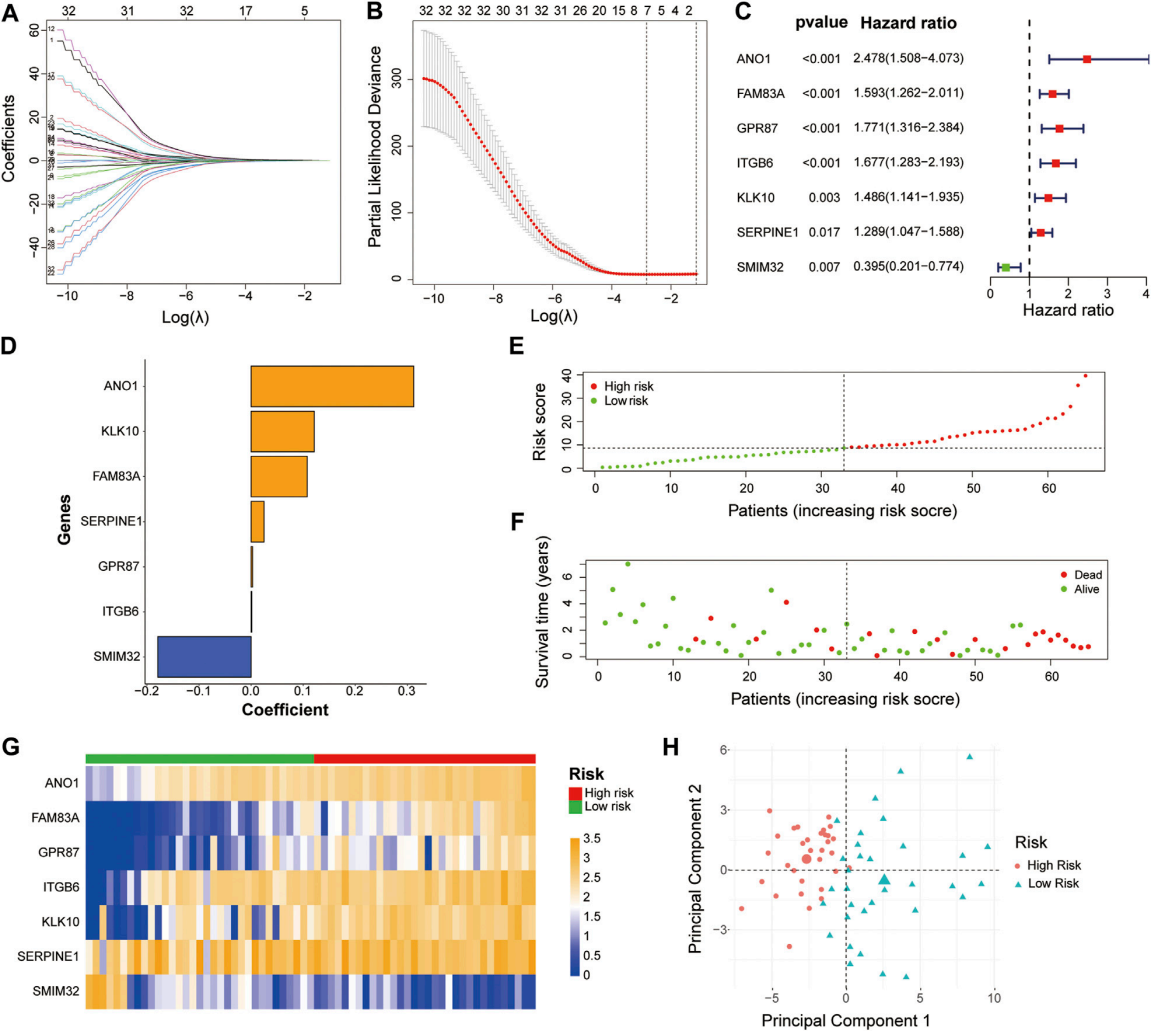
Identification of a 7-gene signature for OS by LASSO regression analysis in the TCGA-PAAD cohort **(A, B)** The LASSO regression was performed to calculate the minimum criteria. The prognostic ability **(C)** and corresponding coefficients **(D)** of the seven liver-metastasis-related genes. Distributions of risk scores based on the liver-metastasis-related prognostic signature **(E)** and survival status **(F)** of the PAAD patients in the TCGA dataset **(G)** Heatmap of the associations between the expression levels of the seven liver-metastasis-related genes and the risk score in the TCGA dataset **(H)** Principal component analysis was performed to assess the difference between the low- and high-risk groups. *OS*, overall survival; *LASSO*, Least absolute shrinkage, and selection operator; *TCGA*, The Cancer Genome Atlas; *PAAD*, pancreatic adenocarcinoma.

To build the LM-PS for forecasting the OS of PAAD patients, we included the expression of the 45 liver-metastasis-related genes into the univariate Cox regression analysis and identified 33 genes significantly associated with OS (Supplementary Table S1). Subsequently, based on the 33 prognostic genes, the LASSO Cox analysis (Figures 2A,B) was performed to construct a seven-gene LM-PS containing anoctamin-1 (ANO1), family with sequence similarity 83, member A (FAM83A), G protein-coupled receptor 87 (GPR87), integrin beta-6 (ITGB6), kallikrein-10 (KLK10), serine protease inhibitor, clade E member 1 (SERPINE1), and small integral membrane protein 32 (SMIM32). The detailed information and the prognostic ability of the seven liver-metastasis-related genes were presented in Table 2 and Figure 2C, respectively. Next, the risk score for predicting OS was calculated for each patient in the training dataset based on the expression and coefficients (Figure 2D) of the seven liver-metastasis-related genes. Then the patients were separated into low- and high-risk subgroups based on the median value of the risk scores (Figure 2E). The survival status of the patients in the high- and low-risk groups was visualized in Figure 2F showing that the mortality rate post R0 resection was higher in the high-risk group than in the low-risk group. The heatmap showed that the expression of the risk genes (ANO1, FAM83A, GPR87, ITGB6, KLK10, and SERPINE1) were up-regulated, while the expression of the protective gene (SMIM32) was down-regulated (Figure 2G) with the increasing risk score. In addition, the PCA was used to assess the biological difference between low- and high-risk groups based on the expression of the seven liver-metastasis-related genes. The result demonstrated that the low- and high-risk patients were distributed separately in distinct directions (Figure 2H).

**TABLE 2:** Information of the seven genes in the liver-metastasis-related prognostic signature.

Gene symbol	Description	Ensemble ID	Location (GRCh38/hg38)
ANO1	Anoctamin-1	ENSG00000131620	chr11:69,985,907–70,189,530
FAM83A	Family with sequence similarity 83	ENSG00000147689	chr8:123,178,960–123,210,079
GPR87	G protein-coupled receptor 87	ENSG00000138271	chr3:151,294,086–151,316,820
ITGB6	Integrin beta-6	ENSG00000115221	chr2:160,099,666–160,200,313
KLK10	Kallikrein-10	ENSG00000129451	chr19:51,012,739–51,020,175
SERPINE1	Serine protease inhibitor, clade E member 1	ENSG00000106366	chr7:101,127,104–101,139,247
SMIM32	Small integral membrane protein 32	ENSG00000271824	chr5:136,191,468–136,193,162

### Prognostic Analysis of the Seven Liver-Metastasis-Related Genes and the LM-PS

**FIGURE 3:**
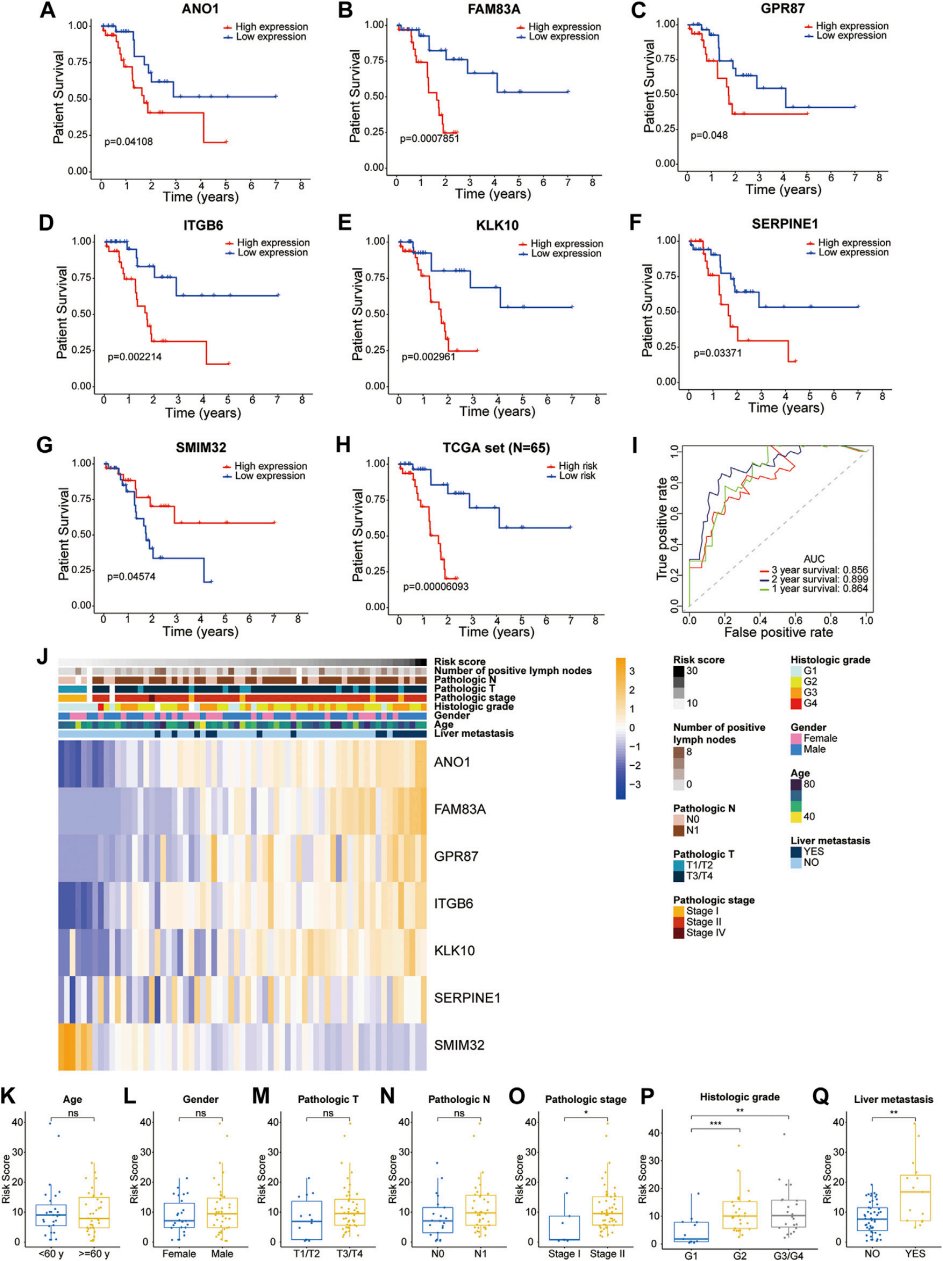
Prognostic analysis of the seven liver-metastasis-related genes and the LM-PS **(A–G)** Kaplan-Meier curves showed that high expression of ANO1, FAM83A, GPR87, ITGB6, KLK10, SERPINE1, and low expression of SMIM32 were significantly associated with worse OS in the TCGA-PAAD dataset **(H)** Kaplan-Meier curves showed that the high-risk subgroup had worse OS than the low-risk subgroup in the TCGA-PAAD dataset **(I)** ROC curves of LM-PS for predicting the 1-, 2-, and 3-years OS in the TCGA dataset **(J)** Heatmap of the associations between the expression levels of the seven liver-metastasis-related genes and clinicopathological features in the TCGA dataset **(K–Q)** Risk scores were higher in patients with G2 and G3/G4, pathologic stage II, and liver metastasis compared with the corresponding control groups but were not significantly associated with age, gender, pathologic T stage, or pathologic N stage. *OS*, overall survival; *LM-PS*, liver-metastasis-related prognostic signature; *TCGA*, The Cancer Genome Atlas; *PAAD*, pancreatic adenocarcinoma; *ROC*, receiver operating characteristic curves; *, *p* < 0.05; **, *p* < 0.01; ***, *p* < 0.001.

The patients in the training dataset were divided into two groups based on the median expression of each liver-metastasis-related gene. The Kaplan-Meier curves showed that high expression of ANO1, FAM83A, GPR87, ITGB6, KLK10, SERPINE1, and low expression of SMIM32 were significantly associated with worse patient survival (Figures 3A–G). In addition, based on the two different risk groups distinguished by the LM-PS, the Kaplan-Meier curve also showed that patients with high-risk had a significantly lower OS rate than those with low-risk (*p* = 6.093E-05, Figure 3H). The OS predictive ability of the LM-PS was confirmed by the ROC curves in the TCGA cohort with 1-year AUC = 0.864, 2-years AUC = 0.899, and 3-years AUC = 0.856 (Figure 3I).

The distribution of clinicopathological factors with the risk score increasing is shown in Figure 3J. In addition, risk scores were observed to be higher in patients with G2 and G3/G4, pathologic stage II, and liver metastasis compared with the corresponding control groups but were not significantly associated with age, gender, pathologic T stage, or pathologic N stage (Figures 3K–Q).

### The Prognostic Ability of the LM-PS to Predict Liver Metastasis

**FIGURE 4:**
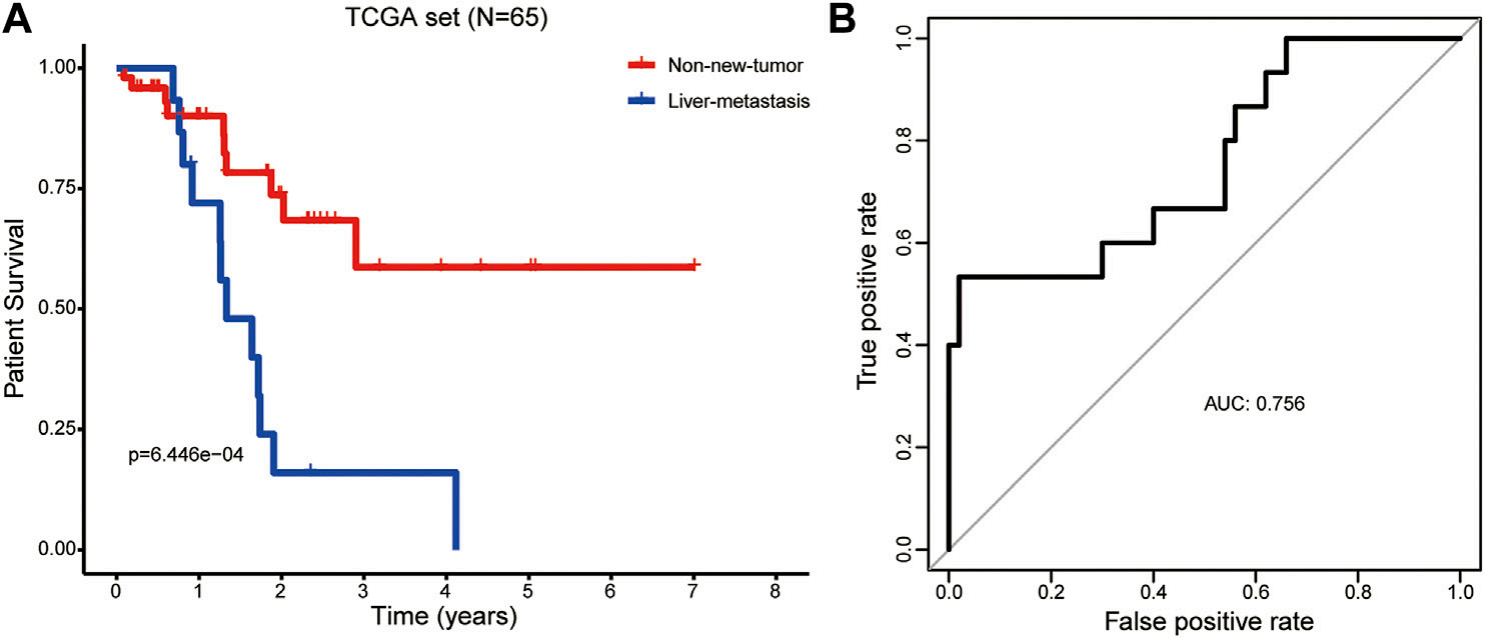
The prognostic ability of the LM-PS to predict liver metastasis in PAAD patients **(A)** PAAD patients with liver metastasis had a significantly worse overall survival than those without new tumor events **(B)** ROC curves of LM-PS for predicting liver metastasis in the training TCGA-PAAD dataset. *LM-PS*, liver-metastasis-related prognostic signature; *PAAD*, pancreatic adenocarcinoma; *ROC*, receiver operating characteristic curves; *TCGA*, The Cancer Genome Atlas.

As expected, the Kaplan-Meier curve showed that the OS rate was remarkably lower in PAAD patients with liver metastasis after R0 resection than those without new tumor events (Figure 4A). This study tried to provide an insight into the factors associated with the liver metastasis of PAAD. The risk score, age, gender, histologic grade, pathologic stage, pathologic T stage, pathologic N stage, and the number of positive lymph nodes were included in univariate logistic regression model analysis. Interestingly, only risk score was significantly associated with liver metastasis [Odds Ratio = 1.176, 95% confidence interval (CI) = 1.061–1.302, *p* = 0.002; Table 3]. The ROC curve confirmed that the risk score harbored a good ability to predict liver metastasis of PAAD in the TCGA cohort (AUC = 0.756, Figure 4B).

**TABLE 3:** Logistic regression analysis of liver metastasis post R0 resection.

Factors	*p*	Or (95% CI)
Risk score	0.002	1.176 (1.061–1.302)
Age	0.571	1.016 (0.961–1.074)
Gender	0.549	0.700 (0.219–2.244)
Histologic grade	0.220	1.718 (0.724–4.077)
Pathologic stage	0.483	1.839 (0.336–10.081)
Pathologic T stage	0.129	5.200 (0.620–43.595)
Pathologic N stage	0.960	1.032 (0.301–3.537)
Number of positive lymph nodes	0.387	1.112 (0.875–1.413)

OR, odds ratio; CI, confidence interval.

### Validation of the LM-PS in the TCGA, GEO and ICGC Datasets

**FIGURE 5:**
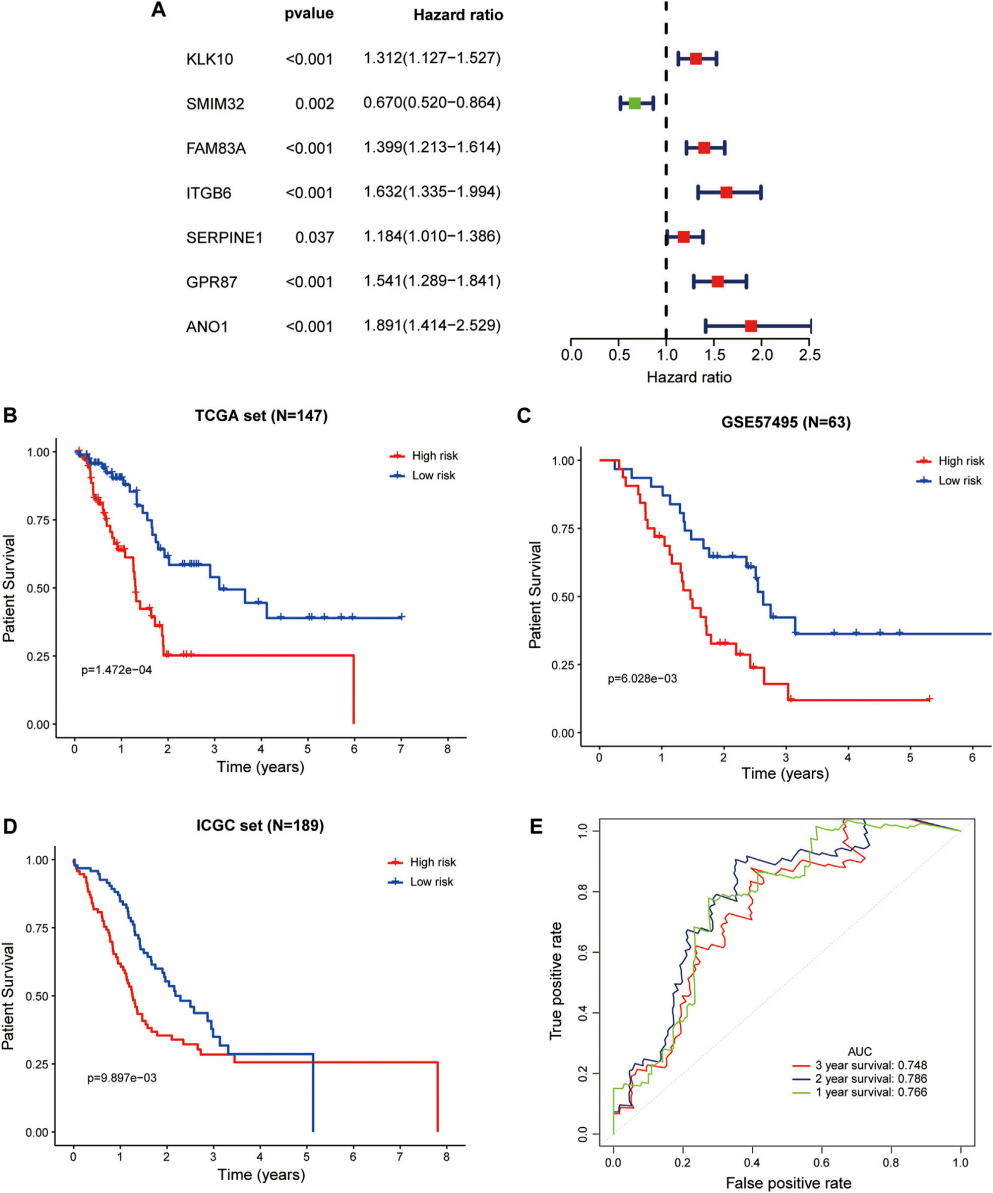
Validation of the LM-PS in the TCGA, GEO, and ICGC Datasets **(A)** Forest plot of the prognostic ability of the seven liver-metastasis-related genes included in the prognostic signature. Kaplan-Meier curves showed that the high-risk group had worse OS than the low-risk group in the whole TCGA **(B)**, GSE57495 **(C)**, and ICGC **(D)** datasets **(E)** Time-dependent ROC curves for the risk score in the whole TCGA dataset for predicting 1-, 2-, and 3-years OS. *TCGA*, The Cancer Genome Atlas; *LM-PS*, liver-metastasis-related prognostic signature; *GEO*, Gene Expression Omnibus; *ICGC*, International Cancer Genome Consortium; *OS*, overall survival.

In the whole TCGA-PAAD cohort, the forest plot showed that high expression of ANO1, FAM83A, GPR87, ITGB6, KLK10, SERPINE1, and low expression of SMIM32 were significantly associated with worse patient survival (Figure 5A). Based on the LM-PS, risk scores were calculated for patients in the whole TCGA-PAAD and GSE57495 cohort and obtained from the SurvExpress website for the ICGC cohort, respectively. The clinicopathological data of the GSE57495 were presented in Supplementary Table S2. Patients in the three datasets were divided into high- and low-risk groups based on the median value of risk scores, respectively. The Kaplan-Meier curves showed that the high-risk patients had significantly lower OS rates than the low-risk patients in the whole TCGA-PAAD, GSE57495, and ICGC datasets (Figures 5B–D). Risk scores and survival status distributions of GSE57495 and ICGC were presented in Supplementary Figures S2A–D. Then the ROC curves were plotted to assess the prognostic values of the risk scores. The 1-, 2-, and 3-years AUCs were 0.766, 0.786, and 0.748 in the whole TCGA dataset, respectively (Figure 5E); 0.601, 0.710, and 0.645 in the GSE57495 dataset, respectively (Supplementary Figure S2E); and 0.719, 0.664, and 0.601 in the ICGC dataset, respectively (Supplementary Figure S2F). These results indicated that the LM-PS harbored a moderate OS predictive ability for PAAD patients.

### The Independent Prognostic Value of LM-PS for PAAD Patients

Univariate and multivariate Cox regression analyses were used to assess the prognostic value of the LM-PS in the TCGA-PAAD dataset. In the training set, the risk score and clinicopathological factors of PAAD patients were included in univariate Cox analysis, and the results showed that risk score was significantly associated with OS [hazard ratio (HR) = 1.132, 95% CI = 1.077–1.190, *p* < 0.001; Table 4]. Furthermore, the multivariate Cox analysis demonstrated that risk score was an independent predictor of OS (HR = 1.126, 95% CI: 1.060–1.197, *p* < 0.001; Table 4).

**TABLE 4:** Univariate and multivariate analyses of clinicopathological factors and LM-PS with OS in the training set.

Factors	Univariate analysis		Multivariate analysis	
	*p*	HR (95% CI)	*p*	HR (95% CI)
Risk score	<0.001	1.132 (1.077–1.190)	<0.001	1.126 (1.060–1.197)
Age	0.056	1.045 (0.999–1.093)	–	–
Gender	0.556	1.300 (0.543–3.113)	–	–
Histologic grade	0.007	2.245 (1.245–4.048)	0.017	2.530 (1.182–5.415)
Pathologic stage	0.042	1.240 (1.008–1.526)	0.055	1.382 (0.993–1.923)
Pathologic T stage	0.023	4.227 (1.225–14.583)	0.196	0.306 (0.051–1.845)
Pathologic N stage	0.016	3.791 (1.278–11.243)	0.069	0.133 (0.015–1.173)
Number of positive lymph nodes	0.025	1.208 (1.025–1.425)	0.005	1.689 (1.168–2.441)

LM-PS, liver-metastasis-related prognostic signature; OS, overall survival; PAAD, pancreatic adenocarcinoma; TCGA, The Cancer Genome Atlas; HR, hazard ratio; CI, confidence interval.

Univariate and multivariate Cox regression analyses were also performed in the whole TCGA-PAAD dataset. The univariate Cox regression analysis showed that risk score was significantly associated with OS (HR = 2.668, 95% CI = 1.761–4.042, *p* < 0.001; Table 5). The multivariate Cox regression analysis further indicated that risk score was an independent predictor of OS in the whole TCGA-PAAD dataset (HR = 2.762, 95% CI: 1.680–4.541, *p* < 0.001; Table 5).

**TABLE 5:** Univariate and multivariate analyses of clinicopathological factors and LM-PS with OS in the whole TCGA-PAAD set.

Factors	Univariate analysis		Multivariate analysis	
	*p*	HR (95% CI)	*p*	HR (95% CI)
Risk score	<0.001	2.668 (1.761–4.042)	<0.001	2.762 (1.680–4.541)
Age	0.014	1.033 (1.007–1.061)	0.264	1.015 (0.989–1.042)
Gender	0.432	1.237 (0.728–2.102)	–	–
Histologic grade	0.001	2.016 (1.354–3.003)	0.092	1.439 (0.943–2.196)
Pathologic stage	0.019	2.242 (1.139–4.411)	0.866	1.111 (0.329–3.755)
Pathologic T stage	0.019	2.608 (1.171–5.811)	0.600	0.736 (0.235–2.312)
Pathologic N stage	0.013	2.321 (1.194–4.512)	0.840	1.090 (0.472–2.515)
Number of positive lymph nodes	0.023	1.076 (1.010–1.146)	0.001	1.181 (1.066–1.309)

LM-PS, liver-metastasis-related prognostic signature; OS, overall survival; PAAD, pancreatic adenocarcinoma; TCGA, The Cancer Genome Atlas; HR, hazard ratio; CI, confidence interval.

### Construction and Validation of a LM-PS-Based Nomogram in the TCGA Dataset

**FIGURE 6:**
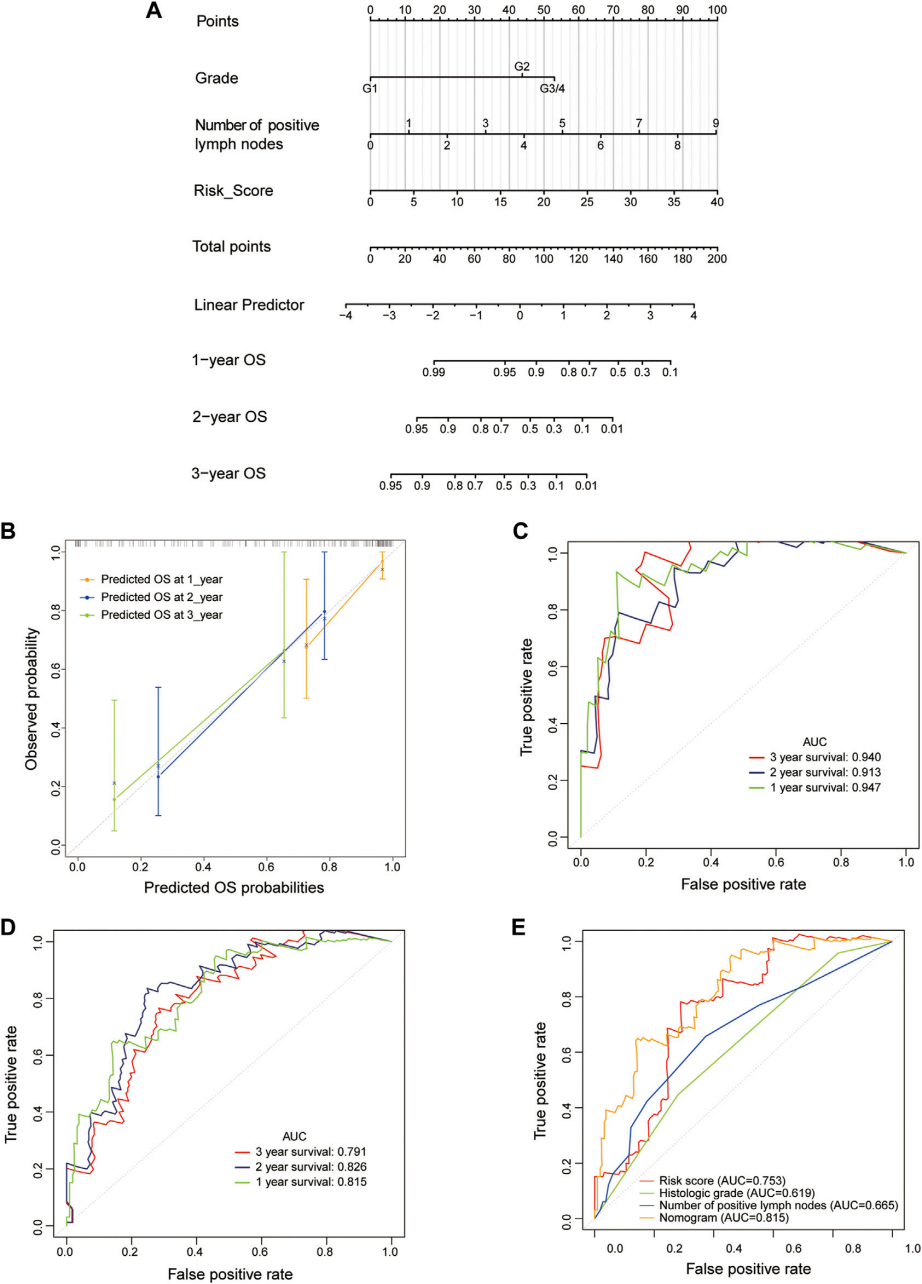
Construction and validation of an LM-PS-based nomogram in the TCGA dataset **(A)** The nomogram is based on risk score, a number of positive lymph nodes, and histologic grade **(B)** The calibration plot of the nomogram for predicting the probability of OS at 1, 2, and 3 years in the TCGA dataset **(C)** Time-dependent ROC curves for the nomogram in the training TCGA-PAAD dataset for predicting 1-, 2-, and 3-years OS **(D)** Time-dependent ROC curves of the nomogram in the whole TCGA dataset for predicting 1-, 2-, and 3-years OS **(E)** ROC curves for the risk score, histologic grade, number of positive lymph nodes, and the nomogram for OS prediction in the whole TCGA-PAAD dataset. *LM-PS*, liver-metastasis-related prognostic signature; *TCGA*, The Cancer Genome Atlas; *OS*, overall survival; *ROC*, receiver operating characteristic curves; *PAAD*, pancreatic adenocarcinoma.

In the training cohort, the risk score, number of positive lymph nodes, and histologic grade were included to establish a clinically applicable quantitative nomogram to predict the OS of PAAD patients (Figure 6A). The calibration plot revealed that the predicted 1-, 2- and 3-years OS using nomogram agreed with the observed OS in the TCGA dataset (Figure 6B). Furthermore, ROC curves were used to assess the prediction accuracy of the nomogram both in the training cohort and the whole TCGA-PAAD cohort. The AUCs values for 1-, 2- and 3-years in PAAD were 0.947, 0.913, and 0.940 in the training cohort, respectively (Figure 6C); and 0.815, 0.826, and 0.791 in the whole TCGA-PAAD cohort, respectively (Figure 6D), indicating high accuracy of the nomogram in the TCGA-PAAD cohort.

Then the ROC curves were also plotted to assess the prognostic values of the nomogram and other independent predictors (risk score, histologic grade, and the number of positive lymph nodes) in the whole TCGA-PAAD dataset. The AUCs of the risk score, histologic grade, number of positive lymph nodes, and the nomogram were 0.753, 0.619, 0.665, and 0.815, respectively, indicating that the inclusion of risk score added to the accuracy of the other two factors included in the nomogram (Figure 6E).

### The Landscape of Immune Infiltration in PAAD

The landscape of immune cell infiltration of PAAD in the TCGA cohort was explored between low- and high-risk groups and between liver-metastasis and no-new-tumor groups using the CIBERSORT algorithm. The violin plots showed that patients in the high-risk subgroup harbored significantly higher prevalence in M0 macrophages (*p* = 0.004) and Eosinophils (*p* = 0.003), but a lower prevalence in naive B cells (*p* = 0.022) than those in the low-risk subgroup (Supplementary Figure S3A). However, no immune cell proportions were observed to be different between non-new-tumor and liver-metastasis subgroups (Supplementary Figure S3B **)**.

### Transcription Factor ‐microRNA Coregulatory Network

**FIGURE 7:**
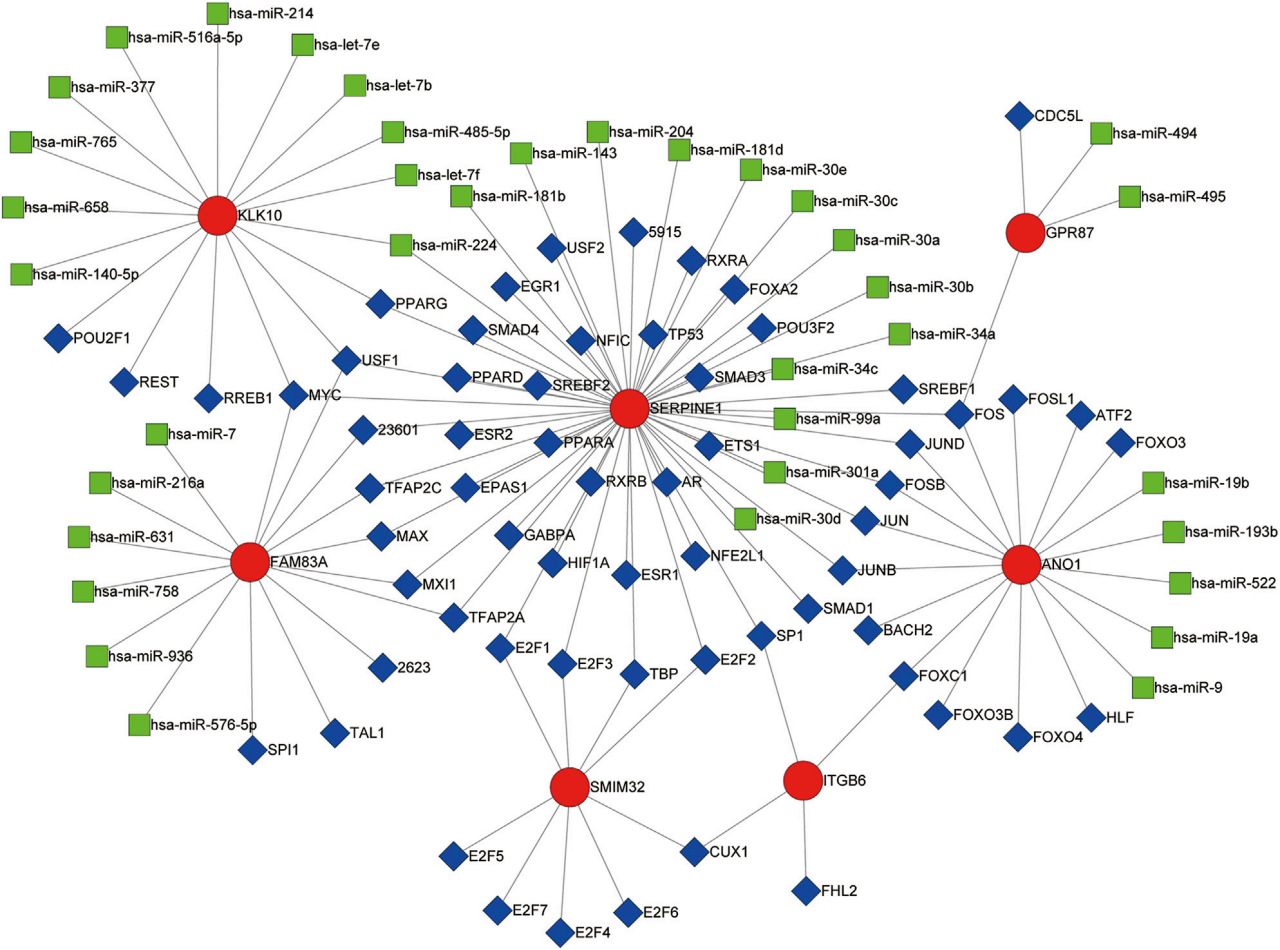
Transcription factor (blue squares)-microRNA (green squares) coregulatory network for the seven liver-metastasis-related prognostic genes (red circles).

The transcription factor‐microRNA coregulatory network based on ANO1, FAM83A, GPR87, ITGB6, KLK10, SERPINE1, and SMIM32 was obtained from NetworkAnalyst. This network contains the seven genes of the LM-PS, 63 transcription factors, and 37 microRNAs, with 86 associations between the LM-PS genes and transcription factors, and 38 associations between the LM-PS genes and microRNAs. As shown in Figure 7, the transcription factors FOS, JUND, USF1, and MYC were observed to regulate three LM-PS genes, the microRNA hsa-miR-224 was observed to modulate two LM-PS genes, and SERPINE1 was the most frequently modulated LM-PS gene. The increase of ANO1, FAM83A, GPR87, ITGB6, and the decrease of SMIM32 were significantly associated with the increase of SERPINE1, while no significant correlation was observed between the expression of KLK10 and SERPINE1 in the TCGA dataset (Supplementary Figures S4A–F).

### Genomic Alterations in PAAD

**FIGURE 8:**
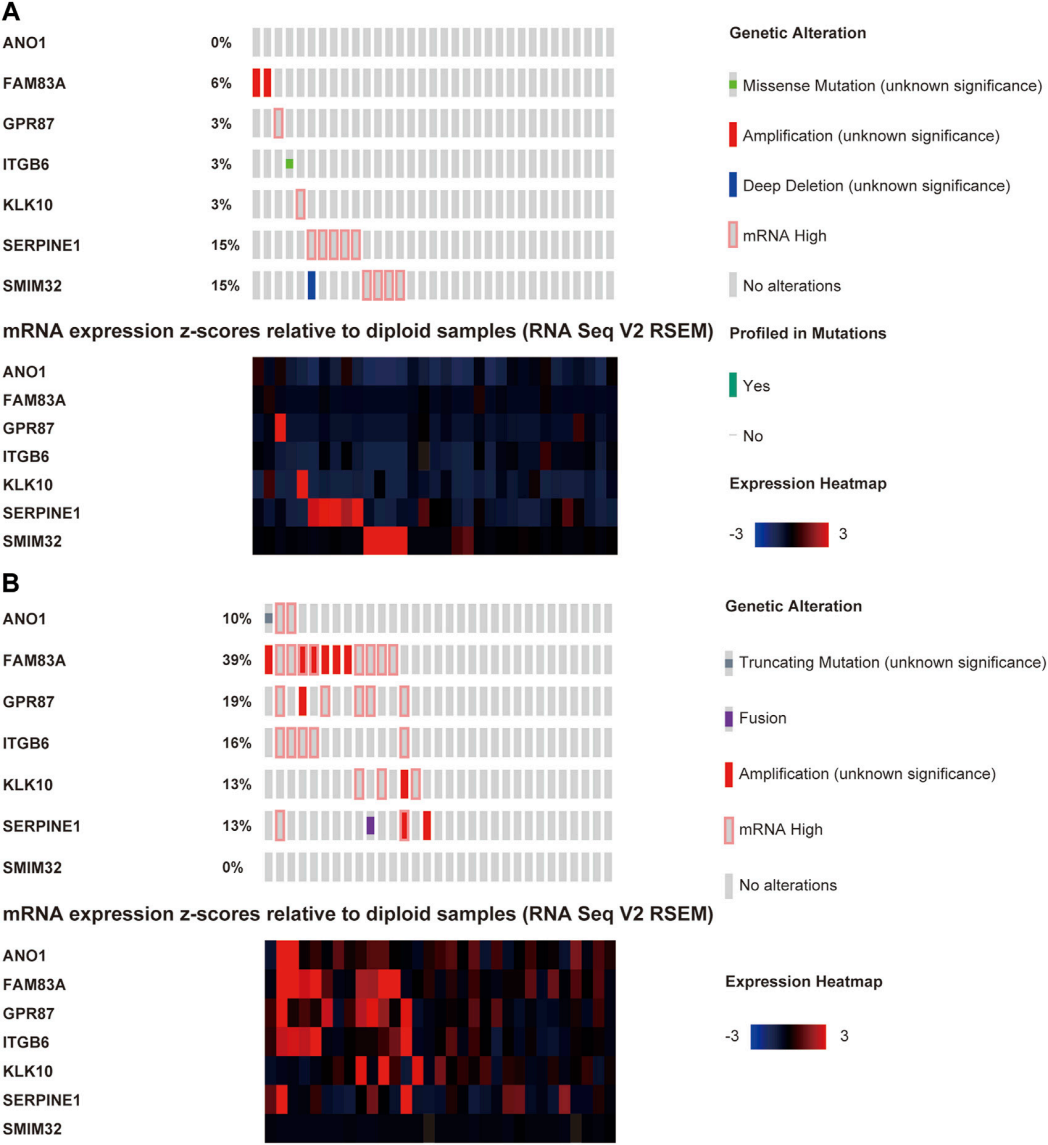
The oncoprints of low- **(A)** and high-risk **(B)** groups.

We explored the genomic alteration for the TCGA-PAAD cohort in this study (data of 64 out of the 65 patients could be found on the cBioPortal website). In this study, 45.3% of the patients (29/64) had at least one LM-PS gene alteration with FAM83A, SERPINE1, and GPR87 being the most frequently altered genes (22%, 14%, and 11%, respectively; Supplementary Figure S5A). The oncoprints of low- and high-risk subgroups are shown in Figures 8A,B. Patients in the high-risk subgroup harbored significantly higher genetic alteration frequency in FAM83A (*p* = 0.002), GPR87 (*p* = 0.037), but lower frequency in SMIM32 (*p* = 0.024) than those in the low-risk subgroup (Supplementary Table S3). In total, no significant difference was observed in OS between the altered and non-altered subgroups (Supplementary Figure S5B). For the individual LM-PS gene alteration, compared to patients with no genetic alteration, patients with alteration of FAM83A and ITGB6 were significantly associated with lower OS (Supplementary Figures S5C,D), while those with alteration of SMIM32 were significantly associated with higher OS (Supplementary Figure S5E). The most frequent mutated genes between the patients with no LM-PS gene altered and those with at least one LM-PS gene altered were also explored. The results showed that the altered subgroup harbored higher enrichment of the V-Ki-ras2 Kirsten Rat Sarcoma Viral Oncogene Homolog (KRAS) (71.43% vs. 48.57%, *p* = 0.0571), Tumor Protein 53 (TP53) (64.29%% vs. 42.86%, *p* = 0.0748), and Cyclin-dependent Kinase Inhibitor 2A (CDKN2A) (28.57% vs. 8.57%, *p* = 0.0405) than the non-altered subgroup (Supplementary Figure S5F).

### Functional Enrichment Analysis of DEGs

**FIGURE 9:**
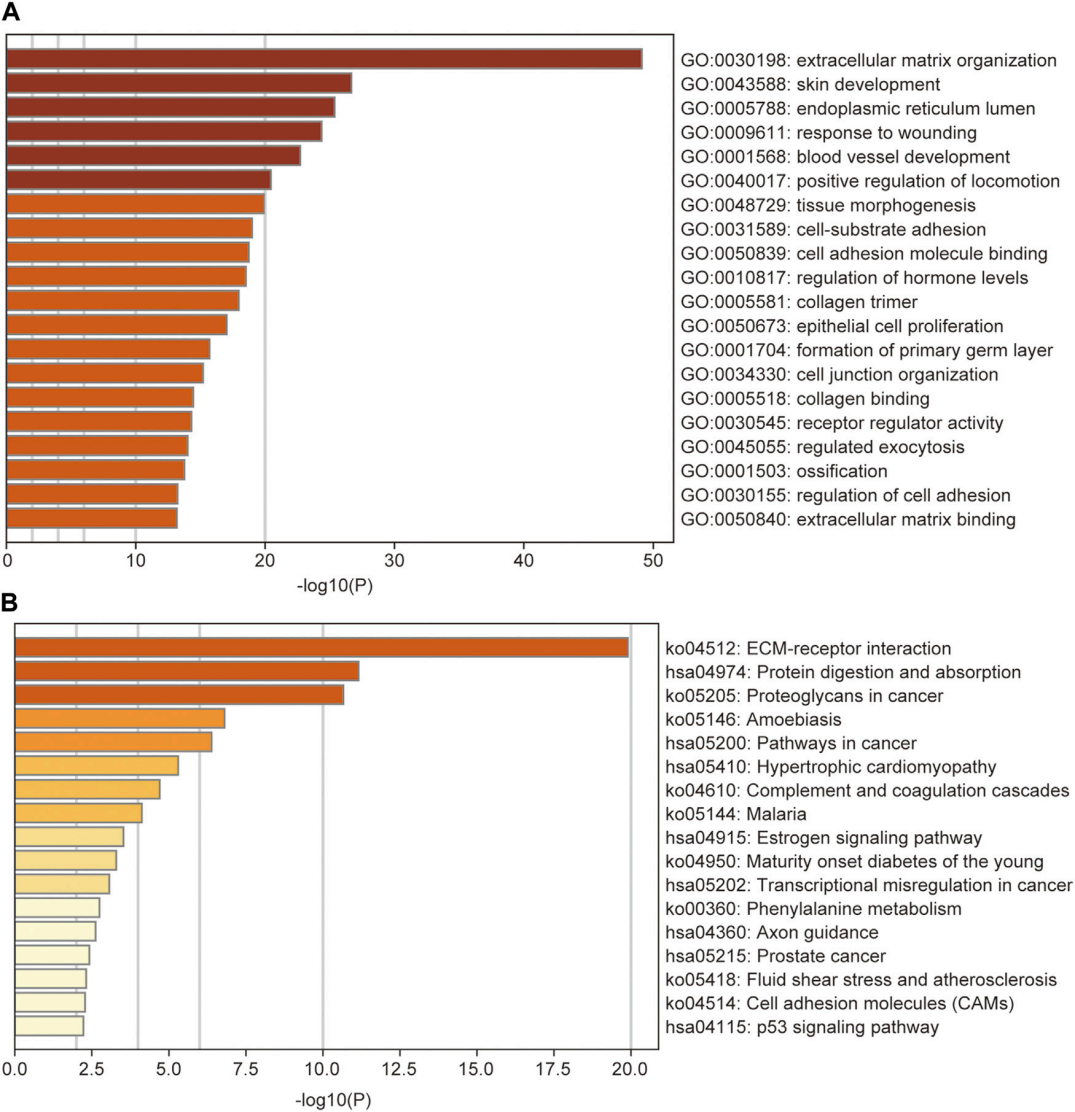
GO **(A)** and KEGG **(B)** analysis of the differentially expressed genes between low- and high-risk groups, colored according to *p*-value. *GO*, Gene Ontology; *KEGG*, Kyoto Encyclopedia of Genes and Genomes Pathway.

GO and KEGG enrichment analyses were performed for the 408 DEGs between high- and low-risk subgroups to identify the most relevant biological processes and pathways. The GO annotations showed that the DEGs enriched in GO terms mainly related to ECM, cell adhesion, and locomotion such as ECM organization, positive regulation of locomotion, cell-substrate adhesion, cell adhesion molecule binding, cell junction organization, collagen binding, regulation of cell adhesion, and ECM binding (Figure 9A). The most enriched KEGG pathway was also ECM-receptor interaction. Additionally, the KEGG enrichment analysis showed that DEGs were enriched in several tumor-related pathways such as proteoglycans in cancer, pathways in cancer, transcriptional misregulation in cancer, prostate cancer, and p53 signaling pathway (Figure 9B).

## Discussion

The current study was mainly based on the TCGA dataset. DEGs between primary PAAD tissues with liver metastasis and those without new tumor events were intersected with DEGs between primary tumor and normal pancreatic tissues, and 45 liver-metastasis-related genes were identified. Thirty-three out of the forty-five DEGs were identified to be significantly associated with the OS of PAAD patients, and seven of them (ANO1, FAM83A, GPR87, ITGB6, KLK10, SERPINE1, and SMIM32) were identified by the LASSO model to construct an LM-PS for predicting the OS of PAAD patients post R0 resection. The up-regulation of ANO1, FAM83A, GPR87, ITGB6, KLK10, and SERPINE1 was significantly associated with poor outcomes of PAAD patients, however, the up-regulation of SMIM32 was protective for the prognosis of PAAD patients. Based on the median value of risk scores generated from the LM-PS, PAAD patients were grouped into the low- and high-risk subgroups and the high-risk subgroup had a significantly lower OS rate than those in the low-risk subgroup. The prognostic value of the LM-PS in the TCGA dataset was validated in the GEO and ICGC datasets. Subsequently, the multivariate Cox analysis and logistic regression analysis confirmed that the LM-PS was a reliable predictor of OS and liver metastasis, respectively. Furthermore, a robust prognostic nomogram based on risk scores, the number of positive lymph nodes, and histologic grade were established to predict 1-, 2-, and 3-years OS for PAAD patients. In addition, immune infiltration analysis showed that only a few kinds of immune cell proportions were different between high- and low-risk subgroups. A transcription factor‐microRNA coregulatory network based on the seven LM-PS genes was constructed for viewing the potential regulatory mechanism of these genes. The cBioPortal was used to give an insight into the impact of genomic alterations on the prognosis of PAAD patients. The genetic alteration frequency in FAM83A, GPR87, and SMIM32 was significantly different between high- and low-risk groups. No significant difference was observed in patient survival between altered and non-altered subgroups. However, compared with those with non-alteration, patients with alterations of FAM83A and ITGB6 were significantly associated with lower OS, while patients with alterations of SMIM32 were significantly associated with higher OS. These results indicate that mutations of FAM83A, ITGB6, and SMIM32 may play important roles in tumor modulation. Additionally, the LM-PS gene altered subgroup was observed to harbor a gene mutation enrichment in KRAS, CDKN2A, and TP53.

The regulatory mechanism of the KRAS, TP53, and CDKN2A has been well discussed. It has been widely acknowledged that KRAS mutation is associated with the poor prognosis of patients with pancreatic cancer [18]. KRAS mutates in approximately 90% of the PDAC cases and promotes the disease mainly by activating the RAS-RAF and PI3K-AKT pathways (an intracellular signaling pathway regulating the cell cycle) [10]. CDKN2A, which encodes protein p16, mutates in more than 90% of the PDAC cases, and the CDKN2A function loss could promote the cell cycle transition from the G1 to S phase [3,19]. TP53, whose mutation was observed in 50–70% PDAC, is one of the critical tumor suppressor genes mutating in the later stages of the disease. The aberrant regulation of TP53 promotes tumor progression by inhibition of apoptosis, regulation of cell cycle, and improvement in cell survival [3].

The modulation of most of the LM-PS genes in this study has been reported to be associated with the progression of pancreatic cancer. A Danish study reported that ANO1 (TMEM16A) was the main component of the Ca^2+^-activated Cl^−^ channel, which was upregulated in PDAC cell lines. ANO1 was reported to be crucial for cell migration but was not significantly associated with cell proliferation in PDAC [20]. Crottès et al. further demonstrated that the high expression of ANO1 promoted pancreatic cancer cell migration by regulating ligand-dependent EGFR signaling pathway and resulted in a low probability of patient survival [21]. These results are consistent with our study, which indicates that ANO1 is a metastasis-related gene in pancreatic cancer.

As a member of the family with sequence similarity 83 (FAM83), FAM83A is, remarkably, up-regulated in PAAD and significantly associated with poor clinical outcomes. Over-expression of FAM83A markedly promotes cancer stem cell traits and chemoresistance by activating the Wnt/β-catenin signaling and TGF-β signaling in pancreatic cancer [22]. The high expression of FAM83A is also essential for the tumorigenesis and the maintaining of MEK/ERK signaling to prevent cell death in pancreatic cancer cells [23]. These results indicate that FAM83A is an important oncogene and a potential biomarker for predicting prognosis and therapeutic targets in pancreatic cancer.

As a G protein-coupled receptor, GPR87 is significantly over-expressed in PAAD tissues and is an independent risk predictor of OS. Elevated GPR87 expression promotes cancer stem cell expansion by regulating the JAK2/STAT3 pathway [24]. A study by Wang et al. demonstrated that up-regulation of GPR87 markedly promoted angiogenesis, proliferation, and resistance to chemotherapy-induced apoptosis of pancreatic cancer by activating the NF-κB signaling pathway [25].

The Kallikreins family are a series of serine proteases modulating the proteolysis scene. They have been reported to be closely correlated with angiogenesis and metastasis [26,27]. Cao et al. reported that the over-expression of KLK10 was observed in the PDAC, especially in those with lymphatic and distant metastasis, and was significantly associated with poor prognosis. The study further revealed that the KLK10 promoted the invasive and metastatic phenotype of PDAC by regulating EMT and FAK-SRC-ERK signaling [28].

As a crucial regulator in the plasminogen activator system, SERPINE1 encodes the plasminogen activator inhibitor and was reported to markedly modulate tumor invasion and proliferation and was negatively associated with the OS of PDAC patients [29,30]. Recently, the study of Akula et al. showed that SERPINE1 was mainly targeted by TP53/miR-34a axis in PDAC [31]. Studies about ITGB6, a member of the ITGB superfamily, in pancreatic carcinogenesis were limited. Only Zhuang et al. reported that the overexpression of ITGB6 in PAAD was significantly associated with the methylation level of CpGs (cg23008083) in promoter region [32]. There has been no study concerning the modulation of SMIM32 in pancreatic cancer, which is a potential protective biomarker and is worth exploring in the future.

The GO enrichment analysis showed that the DEGs were mainly enriched in GO terms associated with ECM, cell adhesion, and locomotion. Additionally, the most enriched KEGG pathway was also ECM-receptor interaction. As a reservoir of numerous signaling molecules, the ECM plays an essential role in the tumor microenvironment and has been found to promote metastasis by the degradation of 500–600 proteases [33–35]. In addition, several tumor-related pathways were also highlighted in the KEGG analysis. Therefore, we hypothesize that some phenotypes of pancreatic cancers harbor the ability to disorder normal ECM organization and cell-substrate adhesion in the tumor microenvironment to promote invasion and metastasis.

Recent studies have demonstrated that a low fraction of naive B cells and a high fraction of M0 macrophages were correlated with the decreased OS of PDAC patients [36,37]. These results were consistent with our results showing that naive B cells were significantly down-regulated, whereas M0 macrophage was significantly up-regulated in the high-risk subgroup. As crucial mediators of the tumor microenvironment, macrophages have been reported to promote angiogenesis, proliferation, and metastasis in solid tumors [38]. The M0 macrophages can be polarized into antitumoral M1 phenotype or protumoral M2 phenotype. The functions and phenotypes of tumor-associated macrophages have been reported to be similar to M2 mononuclear cells [39]. Ye et al. reported that tumor associated macrophages promoted pancreatic cancer by regulating the Warburg effect *via* the CCL18/NF-kB/VCAM-1 axis [40]. However, no significant difference in the proportion of M1 or M2 was observed between low- and high-risk subgroups in our study. Therefore, the mechanism of how M0 macrophages impact the prognosis of pancreatic cancer needs further exploration.

In addition, the transcription factors FOS, JUND, USF1, and MYC were found to regulate three LM-PS genes in the transcription factor‐microRNA coregulatory network. FOS and JUND belong to the Activator Protein 1 family [41]. FOS, which encodes leucine zipper protein, was reported to be over-expressed in pancreatic cancer and was closely correlated with tumor proliferation, differentiation, and apoptosis [42]. JUND was also reported to regulate the progression of pancreatic cancer by activating the tumor suppressor gene RASSF10 [43]. The up-regulation of USF1 has been reported to promote multiple solid tumors such as hepatocellular carcinoma, gastric carcinogenesis, and glioma [44–46]. However, there are few reports on how USF1 modulates the progression of pancreatic cancer, and further studies are needed. As an oncogene that is widely implicated in the pathogenesis of malignancies, MYC is reported to be indispensable in KRAS-driven pancreatic carcinogenesis, and the activation of MYC promotes sporadic liver metastasis in PDAC [47].

The limitations of our study are first, that it was a retrospective study and, therefore, no adjustment could be made for confounding factors that might have influenced the clinical outcomes. Second, batch effects between TCGA and GTEx datasets cannot be eliminated, though they have been minimized and assessed. Third, limited samples of PAAD patients were included in this study, and prospective large sample studies are needed in the future.

In conclusion, our study constructed a seven-gene signature with the prognostic ability to identify PAAD patients with a high-risk of death and liver metastasis after R0 resection. Furthermore, a clinically applicable nomogram incorporating genetic features and clinicopathological factors was established to predict the OS of PAAD patients. The nomogram may facilitate an individual therapeutic strategy, early intervention, and delayed cancer progression in clinical practice.

## Data Availability

The original contributions presented in the study are included in the article/Supplementary Material, further inquiries can be directed to the corresponding authors.

## References

[1] Rahib L Smith BD Aizenberg R Rosenzweig AB Fleshman JM Matrisian LM. Projecting Cancer Incidence and Deaths to 2030: the Unexpected burden of Thyroid, Liver, and Pancreas Cancers in the United States. Cancer Res (2014) 74(11):2913–21. 10.1158/0008-5472.CAN-14-0155 24840647

[2] Are C Chowdhury S Ahmad H Ravipati A Song T Shrikandhe S. Predictive Global Trends in the Incidence and Mortality of Pancreatic Cancer Based on Geographic Location, Socio-Economic Status, and Demographic Shift. J Surg Oncol (2016) 114(6):736–42. 10.1002/jso.24410 27511902

[3] Mizrahi JD Surana R Valle JW Shroff RT. Pancreatic Cancer. The Lancet (2020) 395(10242):2008–20. 10.1016/S0140-6736(20)30974-0 32593337

[4] Siegel RL Miller KD Fuchs HE Jemal A. Cancer Statistics, 2021. CA A Cancer J Clin (2021) 71(1):7–33. 10.3322/caac.21654 33433946

[5] Ryan DP Hong TS Bardeesy N. Pancreatic Adenocarcinoma. N Engl J Med (2014) 371(11):1039–49. 10.1056/NEJMra1404198 25207767

[6] Heinemann V Haas M Boeck S. Systemic Treatment of Advanced Pancreatic Cancer. Cancer Treat Rev (2012) 38(7):843–53. 10.1016/j.ctrv.2011.12.004 22226241

[7] Shi H Li J Fu D. Process of Hepatic Metastasis from Pancreatic Cancer: Biology with Clinical Significance. J Cancer Res Clin Oncol (2016) 142(6):1137–61. 10.1007/s00432-015-2024-0 26250876 PMC11819032

[8] Bailey P Chang DK Nones K Johns AL Patch AM Gingras MC. Genomic Analyses Identify Molecular Subtypes of Pancreatic Cancer. Nature (2016) 531(7592):47–52. 10.1038/nature16965 26909576

[9] Witkiewicz AK McMillan EA Balaji U Baek G Lin W-C Mansour J. Whole-exome Sequencing of Pancreatic Cancer Defines Genetic Diversity and Therapeutic Targets. Nat Commun (2015) 6:6744. 10.1038/ncomms7744 25855536 PMC4403382

[10] Buscail L Bournet B Cordelier P. Role of Oncogenic KRAS in the Diagnosis, Prognosis and Treatment of Pancreatic Cancer. Nat Rev Gastroenterol Hepatol (2020) 17(3):153–68. 10.1038/s41575-019-0245-4 32005945

[11] Jonckheere N Auwercx J Hadj Bachir E Coppin L Boukrout N Vincent A. Unsupervised Hierarchical Clustering of Pancreatic Adenocarcinoma Dataset from TCGA Defines a Mucin Expression Profile that Impacts Overall Survival. Cancers (2020) 12(11):3309. 10.3390/cancers12113309 PMC7697168 33182511

[12] Karasinska JM Topham JT Kalloger SE Jang GH Denroche RE Culibrk L. Altered Gene Expression along the Glycolysis-Cholesterol Synthesis Axis Is Associated with Outcome in Pancreatic Cancer. Clin Cancer Res (2020) 26(1):135–46. 10.1158/1078-0432.CCR-19-1543 31481506

[13] Zhou C Zhao Y Yin Y Hu Z Atyah M Chen W. A Robust 6-mRNA Signature for Prognosis Prediction of Pancreatic Ductal Adenocarcinoma. Int J Biol Sci (2019) 15(11):2282–95. 10.7150/ijbs.32899 31595147 PMC6775308

[14] Venkat S Tisdale AA Schwarz JR Alahmari AA Maurer HC Olive KP. Alternative Polyadenylation Drives Oncogenic Gene Expression in Pancreatic Ductal Adenocarcinoma. Genome Res (2020) 30(3):347–60. 10.1101/gr.257550.119 32029502 PMC7111527

[15] Eisenberg E Levanon EY. Human Housekeeping Genes, Revisited. Trends Genet (2013) 29(10):569–74. 10.1016/j.tig.2013.05.010 23810203

[16] Cerami E Gao J Dogrusoz U Gross BE Sumer SO Aksoy BA. The cBio Cancer Genomics Portal: An Open Platform for Exploring Multidimensional Cancer Genomics Data: Figure 1. Cancer Discov (2012) 2(5):401–4. 10.1158/2159-8290.CD-12-0095 22588877 PMC3956037

[17] Kosti I Jain N Aran D Butte AJ Sirota M. Cross-tissue Analysis of Gene and Protein Expression in Normal and Cancer Tissues. Sci Rep (2016) 6:24799. 10.1038/srep24799 27142790 PMC4855174

[18] Ogura T Yamao K Hara K Mizuno N Hijioka S Imaoka H. Prognostic Value of K-Ras Mutation Status and Subtypes in Endoscopic Ultrasound-Guided fine-needle Aspiration Specimens from Patients with Unresectable Pancreatic Cancer. J Gastroenterol (2013) 48(5):640–6. 10.1007/s00535-012-0664-2 22983505

[19] Kamisawa T Wood LD Itoi T Takaori K. Pancreatic Cancer. The Lancet (2016) 388(10039):73–85. 10.1016/S0140-6736(16)00141-0 26830752

[20] Sauter DRP Novak I Pedersen SF Larsen EH Hoffmann EK. ANO1 (TMEM16A) in Pancreatic Ductal Adenocarcinoma (PDAC). Pflugers Arch - Eur J Physiol (2015) 467(7):1495–508. 10.1007/s00424-014-1598-8 25163766 PMC4464647

[21] Crottès D Lin Y-HT Peters CJ Gilchrist JM Wiita AP Jan YN. TMEM16A Controls EGF-Induced Calcium Signaling Implicated in Pancreatic Cancer Prognosis. Proc Natl Acad Sci USA (2019) 116(26):13026–35. 10.1073/pnas.1900703116 31182586 PMC6600921

[22] Chen S Huang J Liu Z Liang Q Zhang N Jin Y. FAM83A Is Amplified and Promotes Cancer Stem Cell-like Traits and Chemoresistance in Pancreatic Cancer. Oncogenesis (2017) 6(3):e300. 10.1038/oncsis.2017.3 28287611 PMC5533946

[23] Parameswaran N Bartel CA Hernandez-Sanchez W Miskimen KL Smigiel JM Khalil AM. A FAM83A Positive Feed-Back Loop Drives Survival and Tumorigenicity of Pancreatic Ductal Adenocarcinomas. Sci Rep (2019) 9(1):13396. 10.1038/s41598-019-49475-5 31527715 PMC6746704

[24] Jiang J Yu C Guo X Zhang H Tian S Cai K. G Protein-Coupled Receptor GPR87 Promotes the Expansion of PDA Stem Cells through Activating JAK2/STAT3. Mol Ther - Oncolytics (2020) 17:384–93. 10.1016/j.omto.2020.01.006 32405536 PMC7210383

[25] Wang L Zhou W Zhong Y Huo Y Fan P Zhan S. Overexpression of G Protein-Coupled Receptor GPR87 Promotes Pancreatic Cancer Aggressiveness and Activates NF-Κb Signaling Pathway. Mol Cancer (2017) 16(1):61. 10.1186/s12943-017-0627-6 28288630 PMC5348802

[26] Kryza T Achard C Parent C Marchand‐Adam S Guillon‐Munos A Iochmann S. Angiogenesis Stimulated by Human Kallikrein‐related Peptidase 12 Acting *via* a Platelet‐derived Growth Factor B‐dependent Paracrine Pathway. FASEB j. (2014) 28(2):740–51. 10.1096/fj.13-237503 24225148

[27] Dong Y Loessner D Irving-Rodgers H Obermair A Nicklin JL Clements JA. Metastasis of Ovarian Cancer Is Mediated by Kallikrein Related Peptidases. Clin Exp Metastasis (2014) 31(1):135–47. 10.1007/s10585-013-9615-4 24043563 PMC3892111

[28] Cao X-Y Zhang X-X Yang M-W Hu L-P Jiang S-H Tian G-A. Aberrant Upregulation of KLK10 Promotes Metastasis *via* Enhancement of EMT and FAK/SRC/ERK axis in PDAC. Biochem Biophysical Res Commun (2018) 499(3):584–93. 10.1016/j.bbrc.2018.03.194 29621546

[29] Botla SK Savant S Jandaghi P Bauer AS Mücke O Moskalev EA. Early Epigenetic Downregulation of microRNA-192 Expression Promotes Pancreatic Cancer Progression. Cancer Res (2016) 76(14):4149–59. 10.1158/0008-5472.CAN-15-0390 27216198

[30] Xiao Y. Construction of a circRNA‐miRNA‐mRNA Network to Explore the Pathogenesis and Treatment of Pancreatic Ductal Adenocarcinoma. J Cel Biochem (2020) 121(1):394–406. 10.1002/jcb.29194 31232492

[31] Akula SM Ruvolo PP McCubrey JA. TP53/miR-34a-associated Signaling Targets SERPINE1 Expression in Human Pancreatic Cancer. Aging (2020) 12(3):2777–97. 10.18632/aging.102776 31986125 PMC7041729

[32] Zhuang H Zhou Z Ma Z Li Z Liu C Huang S. Characterization of the Prognostic and Oncologic Values of ITGB Superfamily Members in Pancreatic Cancer. J Cel Mol. Med. (2020) 24(22):13481–93. 10.1111/jcmm.15990 PMC7701563 33073486

[33] Wolf K Friedl P. Extracellular Matrix Determinants of Proteolytic and Non-proteolytic Cell Migration. Trends Cel Biol (2011) 21(12):736–44. 10.1016/j.tcb.2011.09.006 22036198

[34] Watt FM Huck WTS. Role of the Extracellular Matrix in Regulating Stem Cell Fate. Nat Rev Mol Cel Biol (2013) 14(8):467–73. 10.1038/nrm3620 23839578

[35] Puente XS Sánchez LM Overall CM López-Otín C. Human and Mouse Proteases: a Comparative Genomic Approach. Nat Rev Genet (2003) 4(7):544–58. 10.1038/nrg1111 12838346

[36] Liu R Liao Y-Z Zhang W Zhou H-H. Relevance of Immune Infiltration and Clinical Outcomes in Pancreatic Ductal Adenocarcinoma Subtypes. Front Oncol (2020) 10:575264. 10.3389/fonc.2020.575264 33489882 PMC7815939

[37] Xu C Sui S Shang Y Yu Z Han J Zhang G. The Landscape of Immune Cell Infiltration and its Clinical Implications of Pancreatic Ductal Adenocarcinoma. J Adv Res (2020) 24:139–48. 10.1016/j.jare.2020.03.009 32322419 PMC7171261

[38] DeNardo DG Ruffell B. Macrophages as Regulators of Tumour Immunity and Immunotherapy. Nat Rev Immunol (2019) 19(6):369–82. 10.1038/s41577-019-0127-6 30718830 PMC7339861

[39] Mantovani A Sozzani S Locati M Allavena P Sica A. Macrophage Polarization: Tumor-Associated Macrophages as a Paradigm for Polarized M2 Mononuclear Phagocytes. Trends Immunol (2002) 23(11):549–55. 10.1016/s1471-4906(02)02302-5 12401408

[40] Ye H Zhou Q Zheng S Li G Lin Q Wei L. Tumor-associated Macrophages Promote Progression and the Warburg Effect *via* CCL18/NF-kB/VCAM-1 Pathway in Pancreatic Ductal Adenocarcinoma. Cell Death Dis (2018) 9(5):453. 10.1038/s41419-018-0486-0 29670110 PMC5906621

[41] Wasylishen AR Sun C Chau GP Qi Y Su X Kim MP. Men1 Maintains Exocrine Pancreas Homeostasis in Response to Inflammation and Oncogenic Stress. Proc Natl Acad Sci USA (2020) 117(12):6622–9. 10.1073/pnas.1920017117 32156729 PMC7104337

[42] Wang Y Liu K Ma Q Tan Y Du W Lv Y. Pancreatic Cancer Biomarker Detection by Two Support Vector Strategies for Recursive Feature Elimination. Biomarkers Med (2019) 13(2):105–21. 10.2217/bmm-2018-0273 PMC6737501 30767554

[43] Richter AM Walesch SK Würl P Taubert H Dammann RH. The Tumor Suppressor RASSF10 Is Upregulated upon Contact Inhibition and Frequently Epigenetically Silenced in Cancer. Oncogenesis (2012) 1:e18. 10.1038/oncsis.2012.18 23552700 PMC3412644

[44] Chen B Chen XP Wu MS Cui W Zhong M. Expressions of Heparanase and Upstream Stimulatory Factor in Hepatocellular Carcinoma. Eur J Med Res (2014) 19:45. 10.1186/s40001-014-0045-9 25149140 PMC4237794

[45] Costa L Corre S Michel V Le Luel K Fernandes J Ziveri J. USF1 Defect Drives P53 Degradation during *Helicobacter pylori* Infection and Accelerates Gastric Carcinogenesis. Gut (2020) 69(9):1582–91. 10.1136/gutjnl-2019-318640 31822580 PMC7456735

[46] Wang J Gu J You A Li J Zhang Y Rao G. The Transcription Factor USF1 Promotes Glioma Cell Invasion and Migration by Activating lncRNA HAS2-AS1. Biosci Rep (2020) 40(8):BSR20200487. 10.1042/BSR20200487 32776110 PMC7442972

[47] Hessmann E Schneider G Ellenrieder V Siveke JT. MYC in Pancreatic Cancer: Novel Mechanistic Insights and Their Translation into Therapeutic Strategies. Oncogene (2016) 35(13):1609–18. 10.1038/onc.2015.216 26119937

