# Supplementary material for: Liver-Metastasis-Related Genes are Potential Biomarkers for Predicting the Clinical Outcomes of Patients with Pancreatic Adenocarcinoma
Source: Pathol Oncol Res. 2021 Jul 5;27:1609822. doi: 10.3389/pore.2021.1609822 (PMC8286999; doi:10.3389/pore.2021.1609822)
Supplement: Supplementary file 1 [file DataSheet1.docx]

Supplementary Material

# Supplementary Figures

**
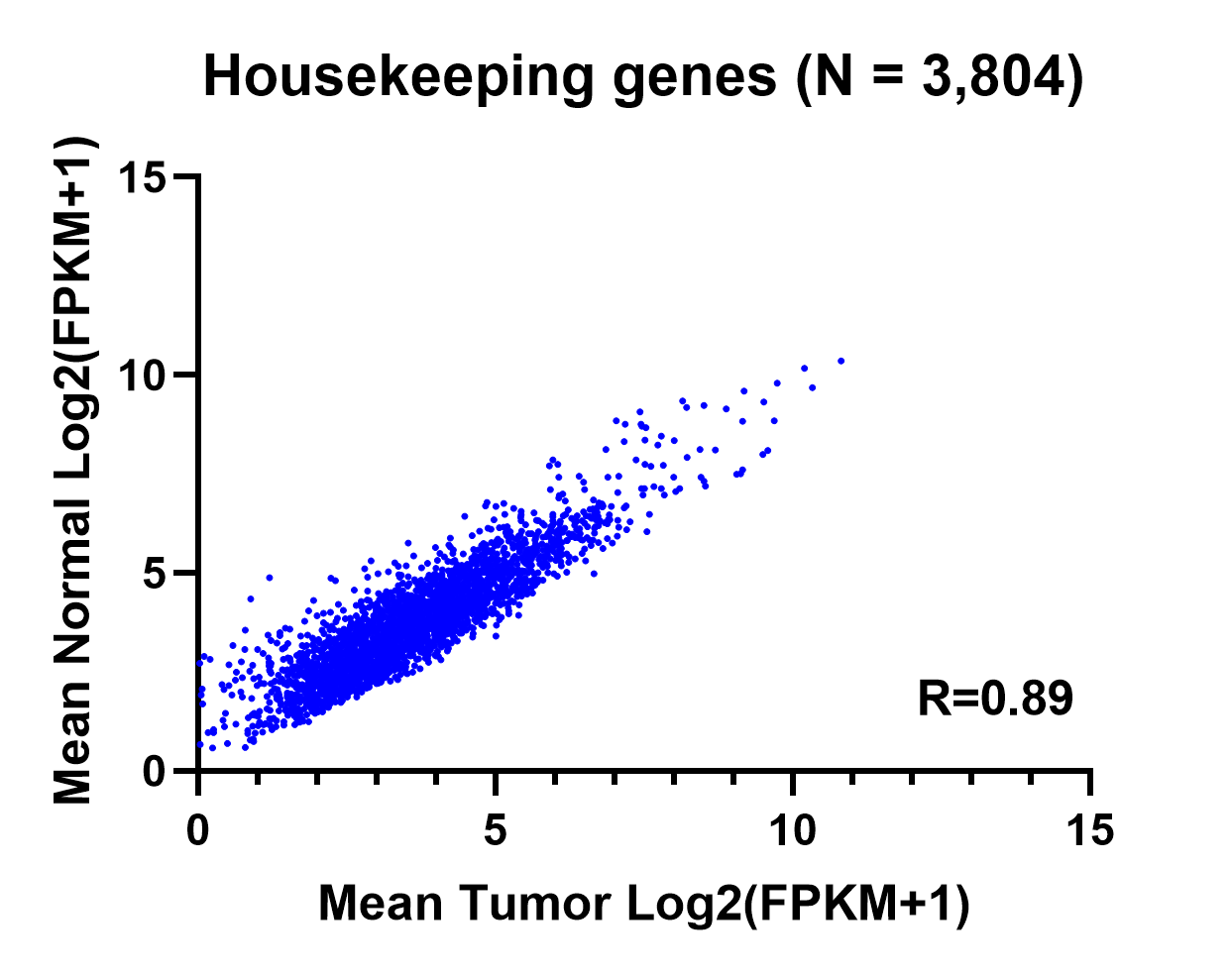
**

**Figure S1|**Mean log_2_(FKPM+1) of 3,804 housekeeping genes between GTEx normal pancreas and TCGA-PAAD tumor tissues. Pearson correlation coefficient (R = 0.89) is presented. *FKPM*, Fragments Per Kilobase of transcript per Million mapped reads; *GTEx*, Genotype-Tissue Expression project; *TCGA*, The Cancer Genome Atlas; *PAAD*, pancreatic adenocarcinoma.

**
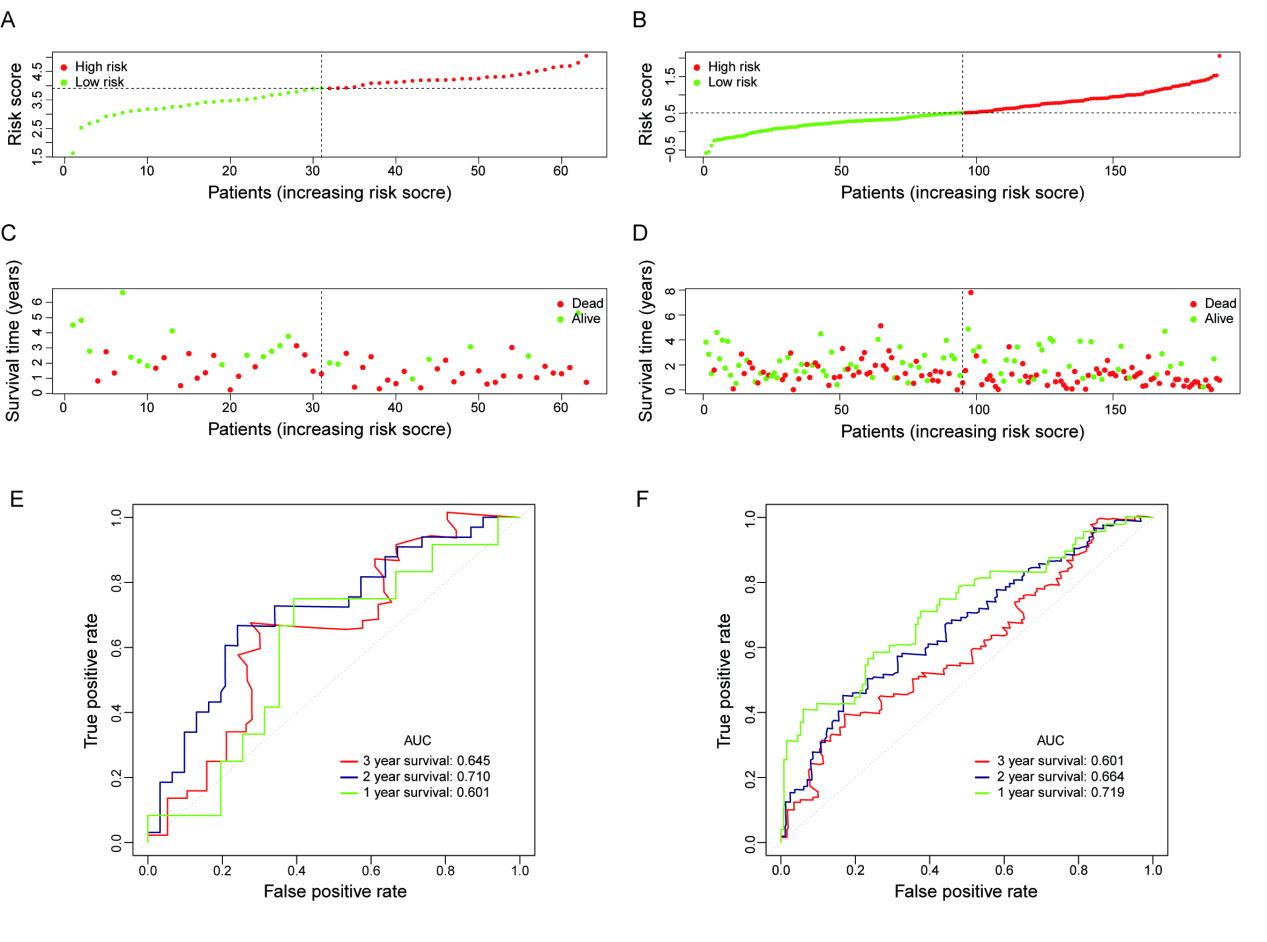
**

**Figure S2|**Distributions of risk scores and survival status of PAAD patients in the GSE57495 dataset **(A,C)** and ICGC dataset **(B,D)**, respectively. ROC curves of LM-PS for predicting the 1-, 2-, and 3-year OS in the GSE57495 **(E)** and ICGC datasets **(F)**, respectively. *PAAD*, pancreatic adenocarcinoma; *ICGC*, International Cancer Genome Consortium; *ROC*, receiver operating characteristic curves; *LM-PS*, liver-metastasis-related genes prognostic signature; *OS*, overall survival.

**
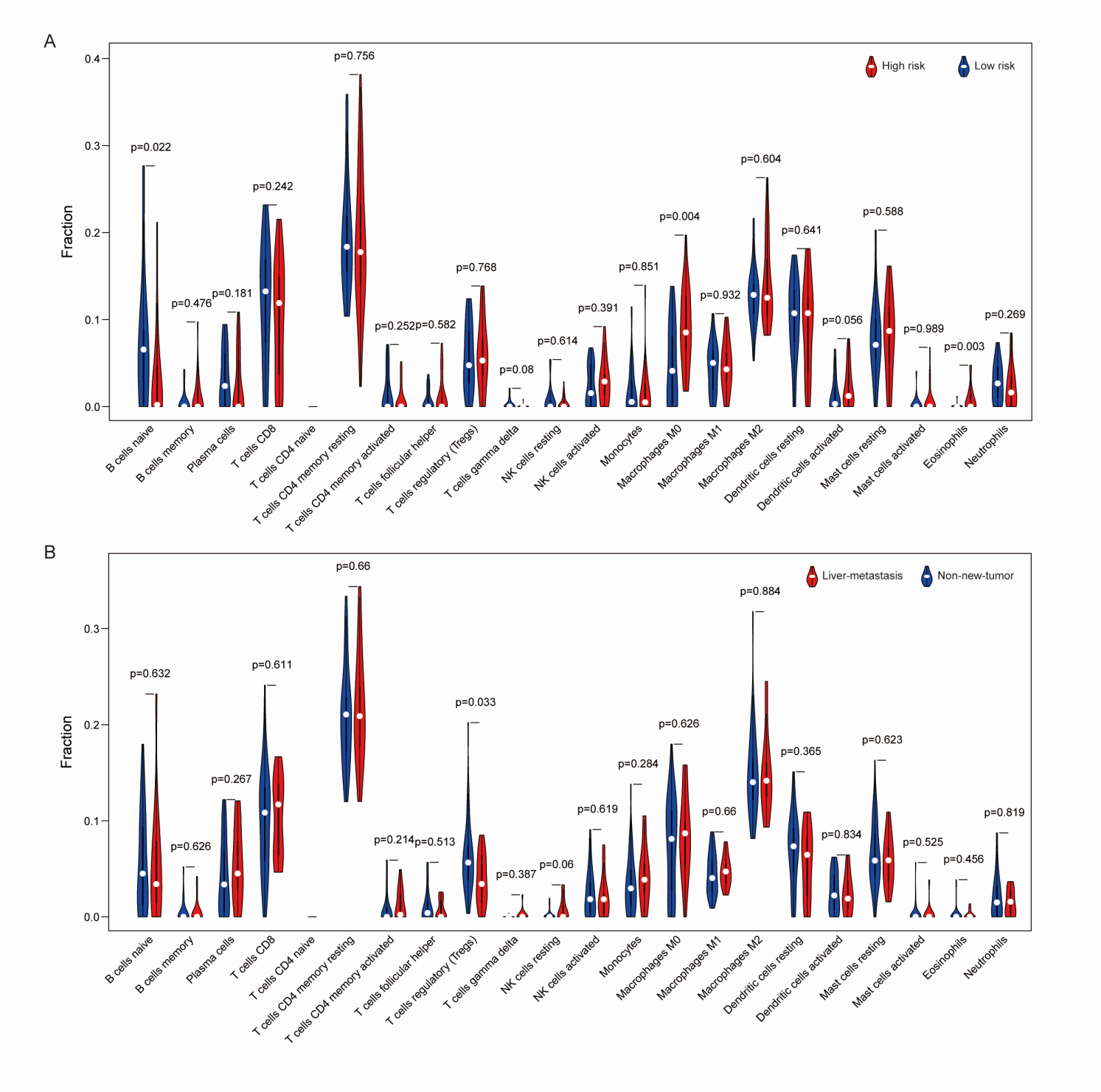
**

**Figure S3|**Violin plots visualizing the differentially infiltrated immune cells between low-risk (blue) and high-risk (red) subgroups **(A)** and between non-new-tumor (blue) and liver-metastasis (red) subgroups **(B)**.

**
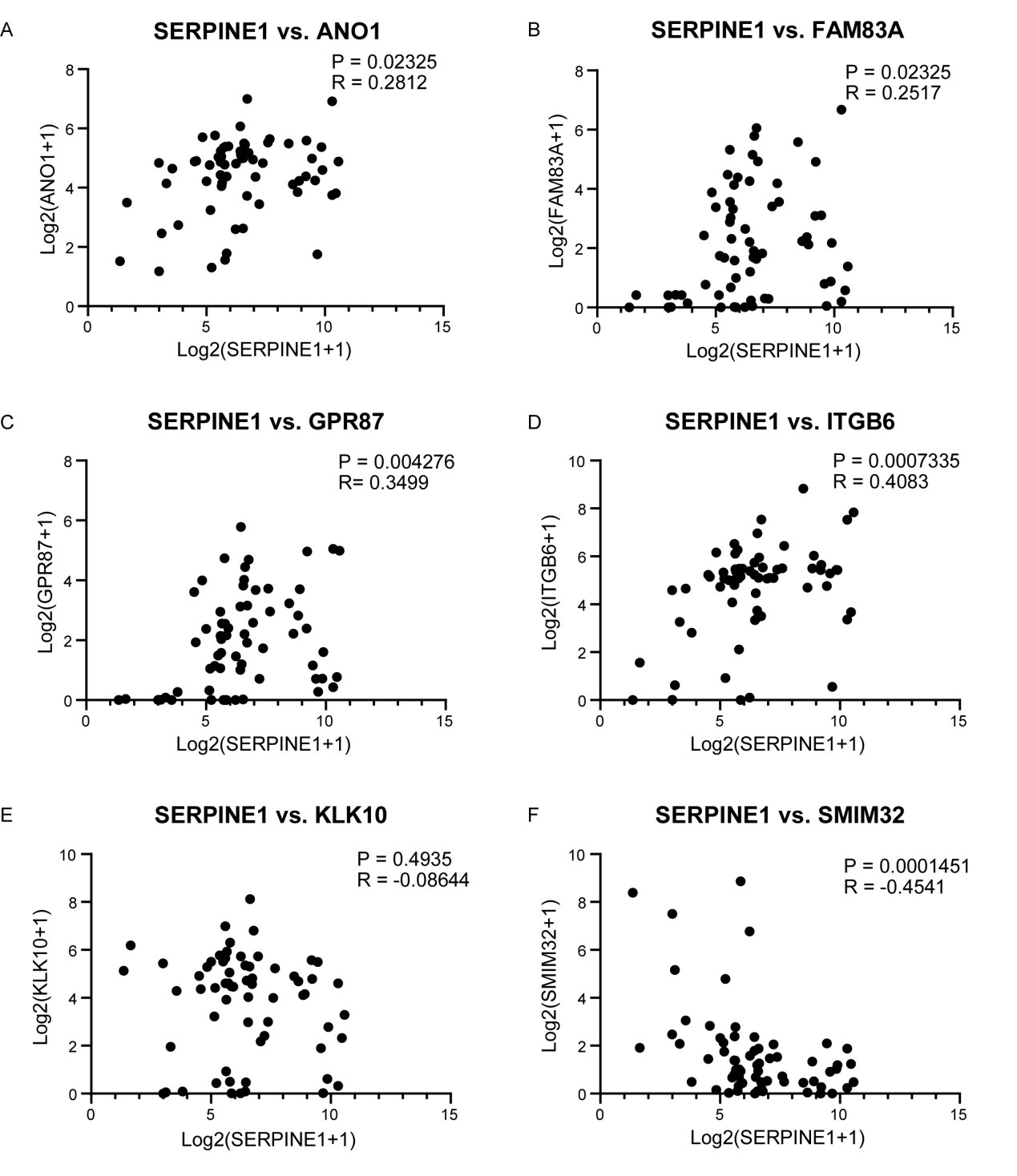
**

**Figure S4|**Correlation analysis between the expressions of ANO1 **(A)**, FAM83A **(B)**, GPR87 **(C)**, ITGB6 **(D)**, KLK10 **(E)**, SMIM32 **(F)**, and SERPINE1 in pancreatic adenocarcinoma.

**
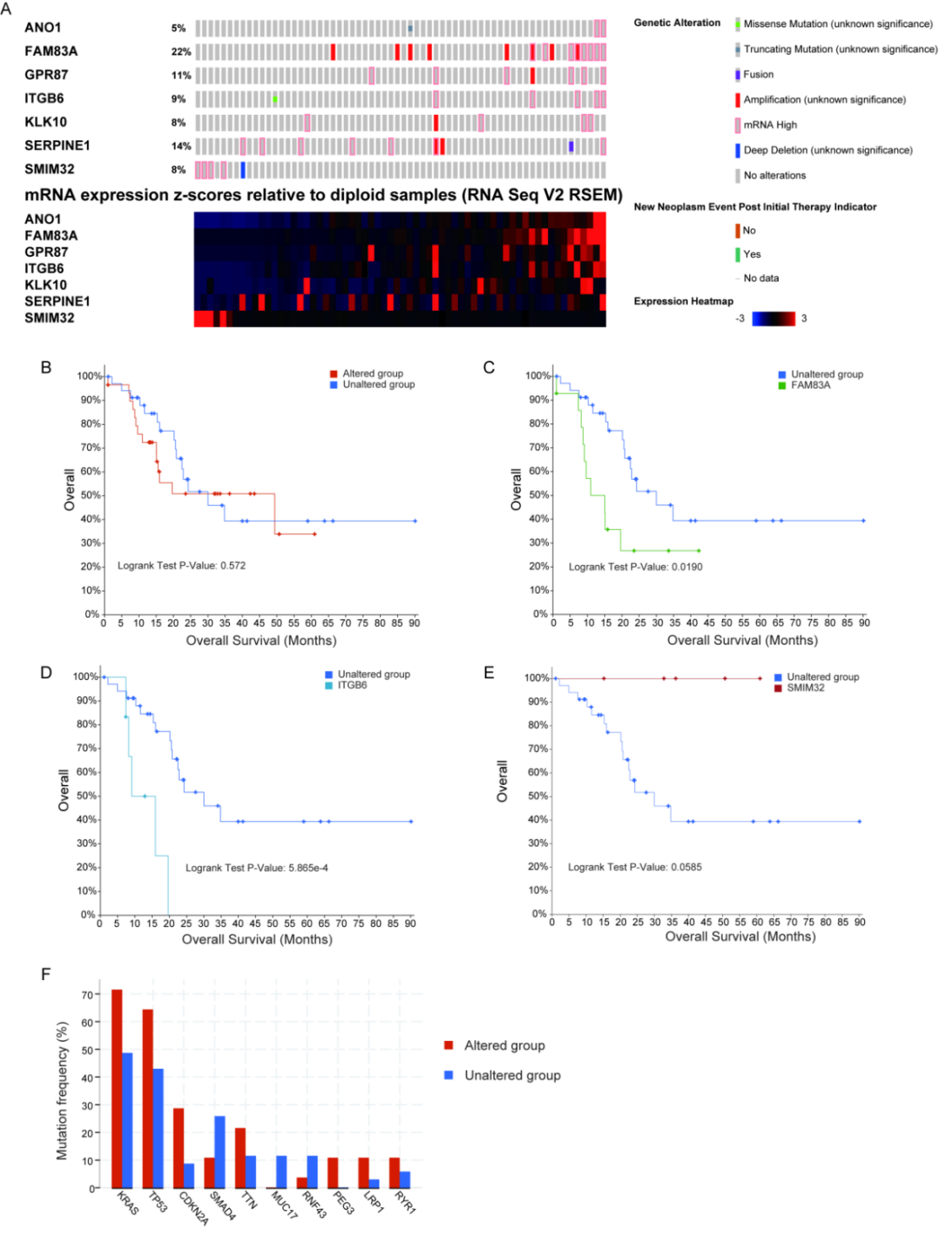
**

**Figure S5|(A)** The cBioPortal oncoprint and heatmap of the 7 LM-PS genes in the TCGA-PAAD cohort. **(B)** OS comparison between patients with LM-PS genes altered (at least with one alteration of amplification/deletion/high mRNA) and those with no LM-PS gene altered in the TCGA-PAAD dataset. **(C)** The OS comparison between PAAD-TCGA patients with alteration of FAM83A and those without alteration. **(D)** The OS comparison between PAAD-TCGA patients with alteration of ITGB6 and those without alteration. **(E)** The OS comparison between PAAD-TCGA patients with alteration of SMIM32 and those without alteration. **(F)** Comparison of the most frequent altered genes between patients with at least one LM-PS gene altered and those with no LM-PS gene altered in the TCGA-PAAD dataset. *LM-PS*, liver-metastasis-related prognostic signature; *TCGA*, The Cancer Genome Atlas; *PAAD*, pancreatic adenocarcinoma; *OS*, overall survival.

# Supplementary Tables

| **Table S1\|**The thirty-three prognostic mRNAs in the univariate Cox regression analysis. | | |
| --- | --- | --- |
| **Gene Symbol** | ***P*** | **HR (95% CI)** |
| ANO1 | 3.45E-04 | 2.478 (1.508-4.073) |
| ANXA1 | 1.13E-03 | 1.743 (1.247-2.435) |
| ANXA8 | 1.21E-03 | 1.798 (1.260-2.564) |
| CDH3 | 3.63E-03 | 1.707 (1.191-2.448) |
| CLIC3 | 8.51E-03 | 1.359 (1.081-1.707) |
| CST6 | 2.25E-02 | 1.265 (1.034-1.548) |
| FAM83A | 8.82E-05 | 1.593 (1.262-2.011) |
| FGFBP1 | 1.47E-02 | 1.322 (1.056-1.654) |
| FN1 | 3.28E-03 | 1.331 (1.100-1.611) |
| GPR87 | 1.63E-04 | 1.771 (1.316-2.384) |
| ITGB4 | 2.27E-03 | 1.732 (1.217-2.465) |
| ITGB6 | 1.58E-04 | 1.677 (1.283-2.193) |
| KLK10 | 3.32E-03 | 1.486 (1.141-1.935) |
| KLK6 | 3.71E-03 | 1.316 (1.093-1.585) |
| KRT6A | 6.08E-04 | 1.317 (1.125-1.542) |
| LAMC2 | 5.46E-03 | 1.348 (1.092-1.664) |
| LYPD3 | 7.65E-03 | 1.546 (1.122-2.129) |
| MELTF | 1.45E-02 | 1.478 (1.080-2.021) |
| MS4A8 | 9.32E-03 | 0.600 (0.408-0.882) |
| PLAU | 8.12E-04 | 1.584 (1.210-2.073) |
| PMEPA1 | 1.51E-02 | 1.431 (1.072-1.910) |
| PTGES | 1.05E-02 | 1.375 (1.077-1.754) |
| S100A2 | 2.34E-03 | 1.314 (1.102-1.566) |
| SCEL | 4.29E-04 | 1.629 (1.242-2.137) |
| SDC4 | 7.18E-03 | 1.577 (1.131-2.198) |
| SEMA3C | 1.09E-03 | 1.656 (1.224-2.241) |
| SERPINE1 | 1.69E-02 | 1.289 (1.047-1.588) |
| SMIM32 | 6.80E-03 | 0.395 (0.201-0.774) |
| SNCG | 1.33E-02 | 1.274 (1.052-1.544) |
| TGM2 | 3.39E-03 | 1.562 (1.159-2.104) |
| TNS4 | 1.44E-02 | 1.306 (1.055-1.616) |
| TRIM29 | 5.55E-03 | 1.461 (1.118-1.910) |
| ZNF185 | 0.000287075 | 1.826 (1.319-2.527) |
| *HR, hazard ratio; CI, confidence interval.* | | |

| **Table S2\|Clinicopathological factors of the PAAD patients in GSE57495 dataset.** | |
| --- | --- |
| **Clinical features** | **n (%)** |
| Survival status, n (%) |  |
| Alive | 21 (33.3) |
| Dead | 42 (66.7) |
| Pathologic stage, n (%) |  |
| Stage I | 1 (1.6) |
| Stage IB | 12 (19.0) |
| Stage IIA | 17 (27.0) |
| Stage IIB | 33 (52.5) |

| **Table S3\|**Comparison of the genomic alteration frequency between the high- and low-risk subgroups. | | | |
| --- | --- | --- | --- |
| **Genes** | **High-risk (n = 31)** | **Low-risk (n = 32)** | ***P*** |
| ANO1 | 3(9.7) | 0 | 0.067 |
| FAM83A | 12(38.7) | 2(6.1) | 0.002 |
| GPR87 | 6(19.4) | 1(3.0) | 0.037 |
| ITGB6 | 5(16.1) | 1(3.0) | 0.072 |
| KLK10 | 4(12.9) | 1(3.0) | 0.141 |
| SERPINE1 | 4(12.9) | 5(15.2) | 0.796 |
| SMIM32 | 0 | 5(15.2) | 0.024 |
